# Bioinformatics insights into the role of GFPT1 in breast invasive carcinoma: implications for tumor prognosis, immune modulation, and therapeutic applications

**DOI:** 10.3389/fgene.2024.1482929

**Published:** 2024-11-22

**Authors:** Jianghui Liang, Xiaolian Deng, Yingyi Zhang, Tianchi Fei, Muzi Ouyang, Chengjie Yu, Yang Xiang, Dongwei Jia, Fangfang Duan

**Affiliations:** ^1^ Shenzhen Key Laboratory for Systems Medicine in Inflammatory Diseases, School of Medicine, Shenzhen Campus of Sun Yat-Sen University, Sun Yat-Sen University, Shenzhen, China; ^2^ Department of Pharmacology, School of Medicine, Shenzhen Campus of Sun Yat-sen University, Shenzhen, China; ^3^ School of Basic Medical Sciences, Shanghai University of Traditional Chinese Medicine, Shanghai, China

**Keywords:** GFPT1, breast invasive carcinoma (BRCA), prognosis, immune infiltration, chemotherapy

## Abstract

**Background:**

Metabolic reprogramming is a hallmark of cancer, including alterations in the hexosamine biosynthesis pathway (HBP). Glutamine-fructose-6-phosphate transaminase 1 (GFPT1) is the key regulatory enzyme in the HBP; however, its role in invasive breast carcinoma remains underexplored.

**Methods:**

This study utilized integrated data from The Cancer Genome Atlas (TCGA) to assess GFPT1 expression in breast cancer (BRCA) patients. Functional enrichment and mutational landscape analyses were performed, along with chemosensitivity predictions. *In vitro* experiments were conducted by silencing GFPT1 in malignant breast epithelial cells to evaluate changes in proliferation, migration, and apoptosis.

**Results:**

Elevated GFPT1 expression was linked to advanced-stage breast cancer and identified as an independent prognostic marker for overall survival (OS). High GFPT1 levels were associated with increased cytoplasmic translation, activation of oncogenic pathways, and infiltration of M2 macrophages. The GFPT1-High group also showed a higher mutational burden, with frequent TP53 mutations. Chemosensitivity analysis revealed increased IC50 values for chemotherapy drugs in this group. GFPT1 silencing led to reduced cell proliferation and migration, along with enhanced apoptosis.

**Conclusion:**

These findings indicate that GFPT1 is a novel prognostic biomarker and a predictive indicator of chemotherapy response in invasive breast carcinoma. GFPT1 influences mRNA translation, cell cycle regulation, and M2 macrophage infiltration, thereby promoting cancer cell proliferation and metastasis.

## 1 Introduction

Breast cancer (BC) remains the most prevalent malignant tumor among women globally and ranks as the second leading cause of cancer-related mortality (Siegel et al., 2021). BC alone accounts for 30% of all cancer diagnoses in women, and among the 14 most common cancers, it has seen the highest increase in mortality rates (Cao et al., 2021; Siegel et al., 2024). Despite advancements in early detection and treatment, leading to a 90% five-year overall survival (OS) rate for BC patients (Wang et al., 2020), the disease becomes incurable once it metastasizes to distant organs such as the brain, bones, liver, and lungs. It is projected to cause over 43,000 deaths globally by 2021 (McAndrew and Finn, 2022). Therefore, the prognosis for individuals diagnosed with invasive breast cancer is a major concern, emphasizing the need for more effective biomarkers and potential new therapeutic options for BC treatment.

Altered metabolism and disrupted cellular energetics are recognized as fundamental hallmarks of all cancers (Akella et al., 2019). Tumor cells reprogram their metabolism by increasing glucose uptake and converting glucose to lactate through fermentation, even in the presence of functioning mitochondria under aerobic conditions (Lin et al., 2020). This increased glycolysis produces intermediates that support cancer cell growth and survival by fueling anabolic pathways for the synthesis of lipids, amino acids, and nucleotides (Ghergurovich et al., 2021). The hexosamine biosynthesis pathway (HBP), a branch of glycolysis, metabolizes 3%–5% of glucose and contributes to the metabolism of carbohydrates, lipids, amino acids, and nucleotides, as well as the production of uridine diphosphate N-acetylglucosamine (UDP-GlcNAc), which leads to aberrant glycosylation in various cancers (de Queiroz et al., 2019; Itkonen et al., 2015; Oikari et al., 2018). HBP influences several aspects of tumor biology, including cell proliferation (Olivier-Van Stichelen et al., 2012), epithelial-to-mesenchymal transition (EMT) (Shaul et al., 2014), stem cell-like properties (26,878,908), cell migration (de Queiroz et al., 2019), and chemotherapy resistance (Liu et al., 2018).

Glutamine-fructose-6-phosphate transaminase (GFPT) is essential for catalyzing the rate-limiting step in hexosamine production and serves as the key regulator of HBP (Oikari et al., 2018). In mammals, there are two GFPT paralogs: GFPT1 and GFPT2, encoded by different genes. GFPT1, located on chromosome 2p13, is ubiquitously expressed and is the predominant form, while GFPT2, located on chromosome 5q34–q35, is mainly expressed in the nervous system (Zhang et al., 2004; Kroef et al., 2022). Recent research has shown that UDP-GlcNAc is associated with GFPT2 expression and promotes hyaluronan synthesis in breast cancer (Oikari et al., 2018). Additionally, epidermal growth factor (EGF) stimulation has been shown to increase GFPT mRNA levels in breast cancer cells (Paterson and Kudlow, 1995), and nicotine-induced O-GlcNAcylation and GFPT expression enhance the EMT and invasion capabilities of breast cancer cells (Zhang et al., 2019). Tissue microarray analyses have indicated elevated GFPT1 expression in triple-negative breast cancer (TNBC) samples (Dong et al., 2016). GFPT1 has been shown to promote breast cancer progression and immune escape through O-glycosylation-modified PD-L1, highlighting its potential role in tumor immune evasion mechanism (Tang et al., 2024). However, the prognostic significance of GFPT1 in invasive breast cancer and its relationship with immune cell infiltration, genetic alterations, and drug sensitivity have not been thoroughly investigated.

The objective of this study was to explore the potential correlation between GFPT1 expression and survival prognosis in breast cancer (BRCA) patients, investigating how elevated GFPT1 levels may predict poor outcomes. Additionally, this study aimed to examine the relationship between GFPT1 expression and immune cell infiltration, genetic alterations, and chemotherapy sensitivity, with the goal of establishing GFPT1 as a novel prognostic biomarker and predictive indicator for chemotherapy response.

## 2 Materials and methods

### 2.1 Data source

Transcriptome profiles, clinical information (including 1,023 BRCA samples and 113 adjacent non-tumor samples), and somatic mutation data (including 1,026 BRCA samples) from breast cancer patients were obtained from TCGA. Additional transcriptome signatures and clinical data were sourced from GSE42568 and GSE22219 datasets in the Gene Expression Omnibus (GEO) database. Protein expression analysis was conducted using data from the Clinical Proteomic Tumor Analysis Consortium (CPTAC), accessed via the UALCAN portal.

### 2.2 GFPT1 expression profiles

The “Gene_DE” module of TIMER2.0 (Tumor Immune Estimation Resource, version 2) was used to evaluate GFPT1 expression levels across various TCGA tumor tissues compared to adjacent normal tissues (Li et al., 2020). For tumors lacking normal tissue samples, such as TCGA-ACC (adrenocortical carcinoma) and TCGA-DLBC (diffuse large B-cell lymphoma), the GEPIA2.0 web server was used to generate box plots to visualize GFPT1 expression differences (Tang et al., 2019). This analysis was performed using a significance threshold of *p* < 0.01 and a minimum log2 fold change of 1, with the expression data log-transformed as log2 (TPM + 1).

### 2.3 Survival prognosis analysis

The Kaplan-Meier plotter, an online survival analysis tool incorporating various microarray datasets, was used to assess the correlation between GFPT1 expression and survival outcomes in cancer patients (Gyorffy, 2021). Multivariate Cox regression analysis was conducted to identify potential prognostic indicators. A nomogram was generated using the R rms package to visually represent prognostic significance. Statistical significance was set at *p* < 0.05, with *p* < 0.001 considered highly significant.

### 2.4 Functional enrichment analysis

The Limma package was used to identify differentially expressed genes (DEGs) between low and high GFPT1 expression groups. DEGs were defined by an adjusted *p*-value <0.05 and log2 (fold change) > 0.5. The ClusterProfiler package was used for Gene Ontology (GO) enrichment analysis and Kyoto Encyclopedia of Genes and Genomes (KEGG) pathway analysis. The gseGO and gseKEGG functions were applied to explore GO terms, covering biological processes, cellular components, and molecular functions, as well as pathway enrichment. Gene Set Enrichment Analysis (GSEA) was performed using GSEA software to identify enriched pathways.

### 2.5 Analysis of tumor-infiltrating immune cells

The ESTIMATE package was used to assess the composition of immune cells (immune score), the degree of stromal cell infiltration (stromal score), the combined stromal-immune score (ESTIMATE score), and the tumor purity of each sample. The CIBERSORT deconvolution algorithm was applied to estimate the proportions of 22 types of tumor-infiltrating immune cells (TIICs) in the TCGA-BRCA cohort. The Vioplot package was then employed to analyze the variations in proportions of these 22 types of TIICs across different groups.

### 2.6 Evaluation of genetic alterations and sensitivity to chemotherapeutic agents

The R package maftools was used to analyze mutations and identify frequently mutated genes within the TCGA-BRCA cohort. The plotmafSummary function was utilized to visualize variant counts per sample, including variant classification, type, SNV class, and overall mutation profiles, highlighting the top 10 most frequently mutated genes. Waterfall plots were generated using the oncoplot function to display gene mutation frequencies. The OncogenicPathways function was used to identify pathways enriched with mutated genes, while the tcgaCompare function compared mutational burdens across different cancer types. Chemosensitivity was predicted using the R package pRRophetic by comparing IC50 values between the GFPT1-High and GFPT1-Low groups.

### 2.7 Cell culture

The Hep3B2.1-7 human hepatocellular carcinoma cell line and breast cancer cell lines (MCF-10A, MCF-7, MDA-MB-231, and T47D) were purchased from Haixing Biosciences (Suzhou, Jiangsu, China). All cell lines were cultured at 37°C in a humidified incubator with 5% CO2 (Thermo Fisher, United States). Cells were maintained in Dulbecco’s Modified Eagle’s Medium (DMEM) supplemented with 10% fetal bovine serum (Zeta Life, AUS), 100 units/mL penicillin, and 100 μg/mL streptomycin.

### 2.8 GFPT1 knockdown, overexpression, and western blot analysis

MDA-MB-231 and MCF-7 cell lines were transfected using Lipofectamine 2000 reagent (Thermo Fisher, United States) with specific siRNAs targeting GFPT1: siRNA-GFPT1#1 (5′-CAA​AGG​CUA​UGA​CUU​CGA​A-3′), siRNA-GFPT1#2 (5′-CAA​GUG​CUG​UCA​UAG​AAC​A-3′), and siRNA-GFPT1#3 (5′-GAA​UCA​UCA​CCA​ACU​ACA​A-3′). A scrambled siRNA was used as a negative control (NC) due to its lack of significant homology to human genome sequences. Final siRNA concentrations were 50 nM for MDA-MB-231 cells and 75 nM for MCF-7 cells, following the manufacturer’s instructions. For GFPT1 overexpression, the negative control (PcDNA3.1), PcDNA3.1 (+)/GFPT-1, were transfected into the cells utilizing Lipofectamine 3,000 (Thermo Fisher, United States) with a final concentration of 11 μg. After 48 h, cells were replenished with complete medium, and western blot analysis confirmed the reduction of GFPT1 protein levels post-transfection.

For western blotting, cell lysates were prepared using radioimmunoprecipitation assay (RIPA) buffer (HUAYUNBIO, HB504A, China) containing protease and phosphatase inhibitors (Roche, United Kingdom). Total protein concentration was determined using the BCA protein assay kit (BEYOTIME BIOTECH INC., P0010S, China). Proteins were separated using 4%–12% Bis–Tris polyacrylamide gels and transferred to PVDF membranes (Millipore, United States). The membranes were washed with TBST, blocked with 5% bovine serum albumin in TBST for 1 h, and incubated with primary antibodies: anti-GFPT1 (1:1,000; 14132-1-AP, Proteintech Group), Anti-GSDMD (1:1,000; PU224937, Abmart) and anti-GAPDH (1:1,000; 60004-1-Ig, Proteintech Group). Membranes were then probed with LI-COR/IRDye 800CW goat anti-rabbit IgG (1:4,000, 926–68071, Proteintech Group) and LI-COR/IRDye 680CW goat anti-mouse IgG (1:4,000, 926–68070, Proteintech Group). Imaging was performed using a CCD camera (Bio-Rad ChemiDoc MP), and band intensities were quantified using a prestained protein ladder (MIKX, Co., Ltd., DB182-01, China).

### 2.9 Assessment of cellular proliferation

Cell growth was assessed using the Cell Counting Kit-8 (MIKX, Co., Ltd., M0856-05, China) following the manufacturer’s procedures. Cells were seeded 8 × 10^3 cells per well in 96-well plates across different treatment groups. The CCK-8 solution was added at designated time intervals, followed by a 2-h incubation at 37°C, and the optical density (OD) was measured at a wavelength of 450 nm for each well using a microplate reader.

### 2.10 EdU staining assay

Cell proliferation was assessed using the EdU staining kit (Beyotime, Shanghai, China) following the manufacturer’s protocol. Cells from different treatment groups were seeded at a density of 3 × 10³ cells per well in 96-well plates and cultured for 48 h. EdU (20 mmol/L) was then added, and the cells were incubated for 2 h. After incubation, cells were fixed with 4% paraformaldehyde for 15 min at room temperature. EdU-positive cells were subsequently analyzed to evaluate the proliferation across treatment groups.

### 2.11 Flow cytometric assessment of cellular apoptosis by employing Annexin V- allophycocyanin (APC)/propidium iodide (PI) staining

Apoptotic cells were identified using an Annexin V-APC/PI apoptosis kit, following the manufacturer’s protocol (MultiSciences, Hangzhou, China). Cells from different treatment groups were seeded at a density of 2 × 10^5 cells per well in 6-well plates and cultured for 48 h. After harvesting, the cells were stained with APC-conjugated Annexin V and PI for 5 min at room temperature in the dark. Following staining, binding buffer was added, and flow cytometry was used to analyze the cell suspensions. Data acquisition (10,000 events per sample) was performed on a fluorescence-activated cell sorting system (CytoFLEX; Beckman Coulter, Brea, CA, United States) using CytoFLEX software.

### 2.12 Cell migration assay

Cells from various treatment groups were seeded at a density of 2 × 10^4 cells per well in the upper chamber (Corning 353,097) with DMEM without fetal bovine serum (FBS). The lower chamber contained DMEM supplemented with 10% FBS. After 48 h of incubation, the cells were fixed with 4% paraformaldehyde, followed by staining with 0.1% crystal violet solution. The number of migrated cells was then manually counted under a microscope.

### 2.13 Statistical analysis

Continuous variables were compared using either Student’s t-test or the Wilcoxon rank-sum test. The Pearson correlation test was used to evaluate the relationships between different sample factors. Survival probabilities were calculated using the Kaplan-Meier method, with differences assessed by the log-rank test. All statistical tests were two-sided, and statistical significance was set at *p* < 0.05.

## 3 Results

### 3.1 GFPT1 expression profiles in different human cancers

**FIGURE 1:**
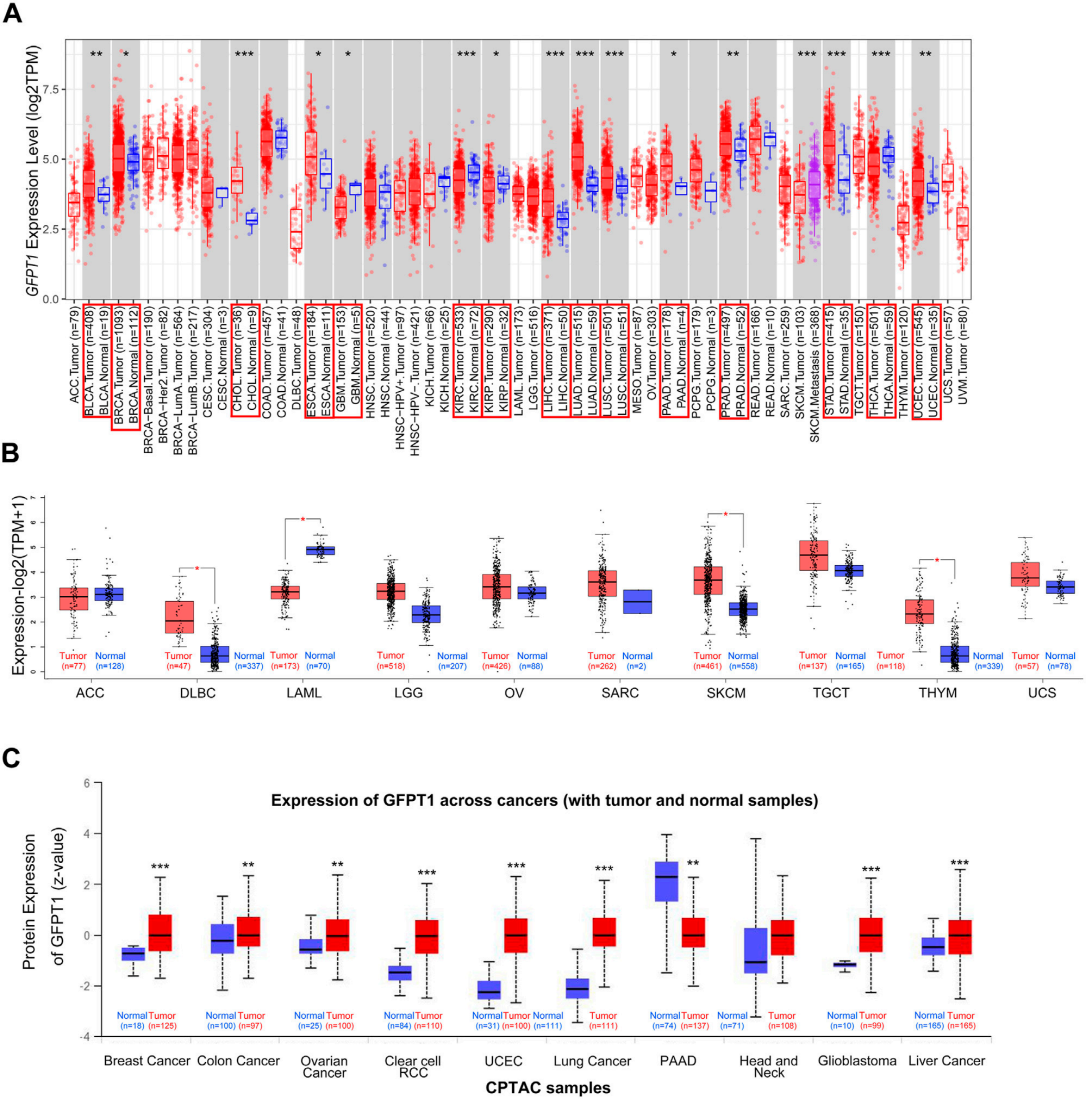
Expression level of GFPT1 in different tumors and adjacent normal tissues. **(A)** The expression status of GFPT1 gene in different tumor types from TCGA database were analyzed by TIMER2.0. Gene expression is measured in log2TPM (TPM, transcripts per million mapped reads). Distributions of gene expression levels are displayed using box plots and the bar represents median expression. Red is for tumors and blue is for normal tissues. The statistical significance computed by the Wilcoxon test is annotated by the number of stars (*p < 0.05; **p < 0.01; ***p < 0.001) and tumor types with statistically significant expression of GFPT1 are indicated by the red boxes. **(B)** For the tumor type of ACC, DLBC, LAML, LGG, OV, SARC, SKCM, TGCT, THYM, and UCS in the TCGA dataset, the corresponding normal tissues of the GTEx database were included as controls. Gene expression is measured in log2 (TPM+1). The box plot data were supplied. Red is for tumors and blue is for normal tissues. *p < 0.05. **(C)** Based on the CPTAC dataset, the expression level of GFPT1 total protein was compared between normal tissues and primary tumor tissues from Breast Cancer, Colon Cancer, Ovarian Cancer, Clear cell RCC, UCEC, Lung Cancer, PAAD, Head and Neck, Glioblastoma, Liver Cancer.

To comprehensively analyze the expression and distribution of GFPT1 in various tumor tissues and adjacent normal tissues, we initially used the TIMER 2.0 tool to examine GFPT1 mRNA expression across multiple cancer types in the TCGA dataset. As shown in Figure 1A, GFPT1 expression was significantly higher in tumor tissues compared to adjacent normal tissues in several cancer types, including BLCA (bladder urothelial carcinoma, *p* = 5.63E-03), BRCA (breast invasive carcinoma, *p* = 2.56E-2), CHOL (cholangiocarcinoma, *p* = 4.07E-06), ESCA (esophageal carcinoma, *p* = 4.99E-02), LIHC (liver hepatocellular carcinoma, *p* = 9.61E-10), LUAD (lung adenocarcinoma, *p* = 5.31E-25), LUSC (lung squamous cell carcinoma, *p* = 3.15E-05), PAAD (pancreatic adenocarcinoma, *p* = 2.50E-2), PRAD (prostate adenocarcinoma, *p* = 8.55E-3), STAD (stomach adenocarcinoma, *p* = 1.80E-08), and UCEC (uterine corpus endometrial carcinoma, *p* = 1.83E-03). In contrast, GFPT1 expression was lower in GBM (glioblastoma multiforme, *p* = 0.020), KIRC (kidney renal clear cell carcinoma, *p* = 3.79E-06), KIRP (kidney renal papillary cell carcinoma, *p* = 0.016), and THCA (thyroid carcinoma, *p* = 7.99E-05) tumor tissues compared to normal tissues (Figure 1A).

To expand this analysis, we incorporated normal tissue samples from the GTEx dataset as controls, comparing GFPT1 mRNA expression between cancerous and normal tissues across additional cancer types, including ACC, DLBC, LAML, LGG, OV, SARC, SKCM, TGCT, THYM, and UCS. Significant increases in GFPT1 expression were observed in DLBC, SKCM, and THYM tumor tissues, while no notable differences were found in ACC, LGG, OV, SARC, TGCT, or UCS samples (Figure 1B). Additionally, GFPT1 expression was lower in LAML tumor tissues compared to normal controls.

To further assess GFPT1 protein expression across different cancer types, we analyzed data from the CPTAC dataset. As shown in Figure 1C, GFPT1 protein levels were elevated in breast cancer, colon cancer, ovarian cancer, clear cell RCC, uterine corpus endometrial carcinoma, lung cancer, glioblastoma, and liver cancer tissues compared to normal tissues. In contrast, pancreatic adenocarcinoma tissues showed lower GFPT1 protein expression compared to normal tissues.

Overall, our pan-cancer analysis revealed increased GFPT1 mRNA and protein levels in BRCA, LIHC, and UCEC tumor tissues compared to normal tissues. Further analysis using the CPTAC database demonstrated a positive correlation between GFPT1 protein levels and advanced tumor stages in BRCA and UCEC, suggesting a potential link between GFPT1 and tumor progression (Supplementary Figure S1A, B). Additionally, higher GFPT1 protein expression was significantly associated with higher tumor grades in UCEC, and GFPT1 levels varied across different molecular subtypes of BRCA (Supplementary Figure S1A, B).

### 3.2 Correlation of GFPT1 levels with patient outcomes in the TCGA-BRCA cohort

**FIGURE 2:**
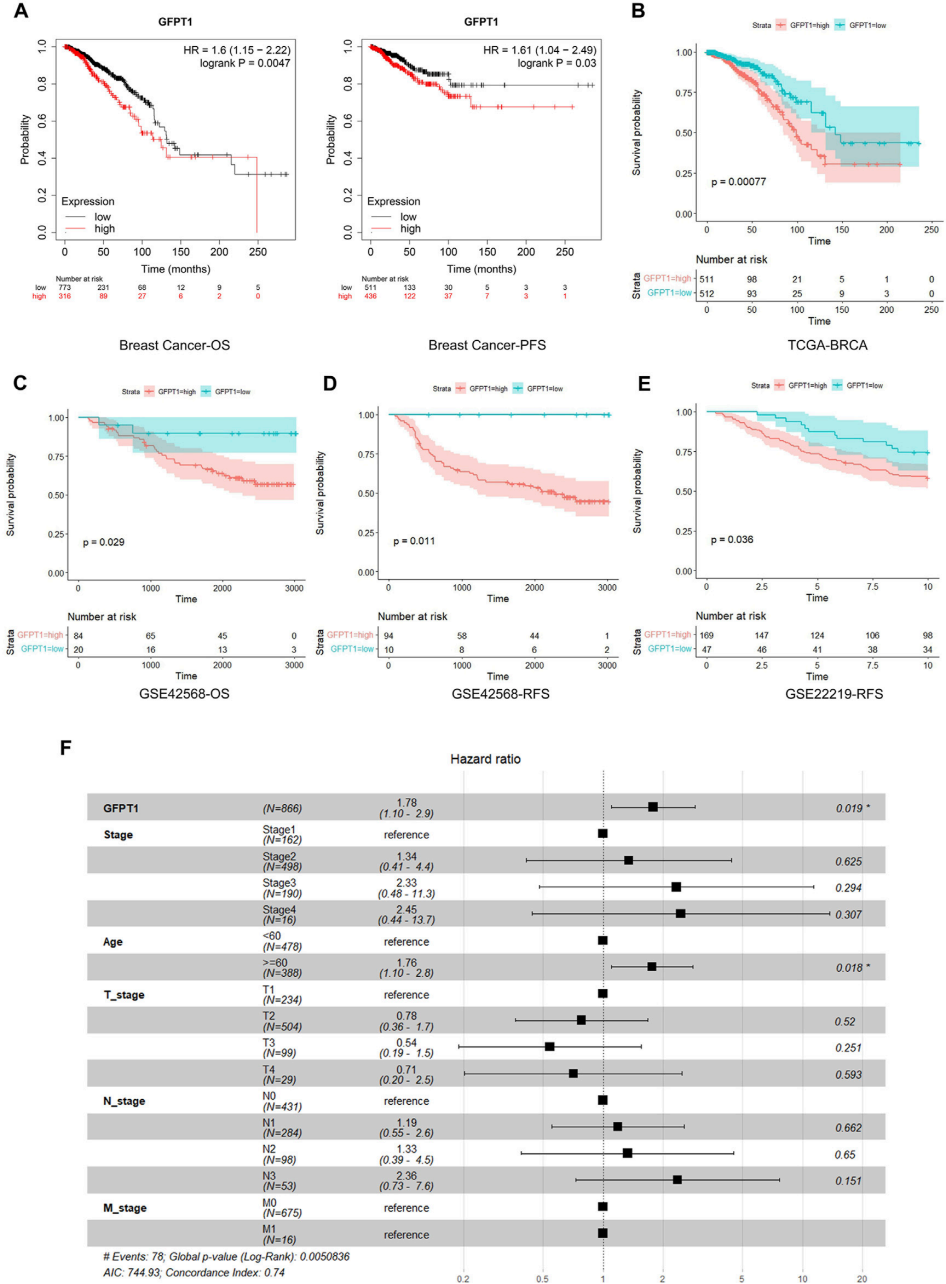
Kaplan-Meier survival analysis for overall survival of breast cancer patients according to the GFPT1 expression. **(A)** The association of GFPT1 expression with Overall survival (OS) and Progression-free survival (PFS) was examined by Kaplan-Meier analysis in breast cancer patients using the online survival analysis software (https://kmplot.com/analysis/). **(B)** The association of GFPT1 expression with OS was examined by Kaplan-Meier analysis in breast cancer patients from TCGA dataset. **(C–D)** Kaplan-Meier survival analysis based on GSE42568 dataset for OS **(C)** and relapse-free survival (RFS) **(D)** respectively. **(E)** Kaplan-Meier survival analysis based on GSE22219 dataset for RFS. **(F)** Multivariate Cox regression analysis of clinicopathological characteristics influencing the OS of breast cancer patients.

We then assessed the correlation between GFPT1 expression and patient survival in BRCA, LIHC, and UCEC using Kaplan-Meier analysis and the log-rank test. An online survival tool (https://kmplot.com/analysis/), which integrates published RNA-seq datasets, revealed a significant association between high GFPT1 expression and reduced overall survival (OS) and progression-free survival (PFS) specifically in breast cancer patients (Figure 2A) (Lanczky and Gyorffy, 2021). However, GFPT1 mRNA levels were not significantly predictive of OS in UCEC patients or relapse-free survival (RFS) in LIHC and UCEC patients (Supplementary Figure S2A, B). These findings suggest that GFPT1 is highly expressed in breast cancer tissues and is associated with a poorer prognosis in breast cancer patients.

Next, we utilized data from TCGA and GEO to further explore the relationship between GFPT1 expression and survival prognosis in breast cancer patients. In the TCGA cohort, elevated GFPT1 expression in tumor tissues was correlated with poorer survival outcomes in breast cancer patients (Figure 2B). Similarly, in the GSE42568 dataset, GFPT1 expression was a significant predictor of OS and RFS in breast cancer patients (Figures 2C, D). Comparable predictive trends for RFS were observed in the GSE22219 dataset (Figure 2E).

To evaluate the prognostic significance of GFPT1 and other clinicopathological factors for OS, we conducted a univariate Cox regression analysis. Significant risk factors for OS in breast cancer patients from the TCGA cohort included age (*p* = 1.2E-03), cancer stage (*p* = 1.6E-05), lymph node stage (*p* = 6.6E-07), distant metastasis stage (*p* = 0.005), and GFPT1 expression (*p* = 1.5E-04) (Supplementary Figure S3). Multivariate Cox analysis, adjusting for covariates, confirmed that both age (*p* = 0.018) and GFPT1 expression (*p* = 0.019) were independent predictors of OS in TCGA-BRCA patients (Figure 2F). In conclusion, GFPT1 serves as an independent prognostic marker in breast cancer patients, significantly associated with poorer survival outcomes.

### 3.3 Prognostic nomogram model for the overall survival analysis of breast cancer patients

**FIGURE 3:**
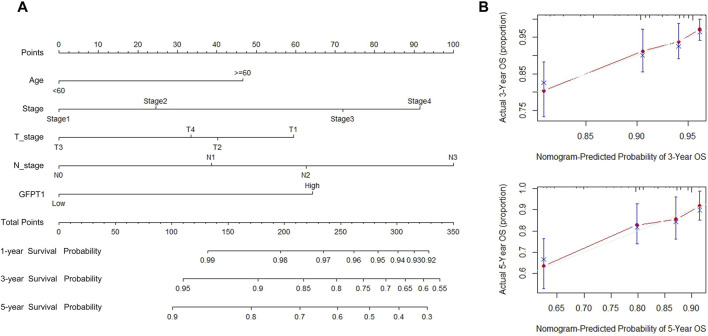
Establishment and validation of the prognostic nomogram for the prediction of survival in breast cancer. **(A)** OS nomogram generation by multivariate Cox regression for predicting the survival of patients with breast cancer. This nomogram was based on Age (<60,≥60), Stage (Stage I, II, III, IV), T_stage (T1, T2, T3), N_stage (N0, N1, N2, N3), and GFPT1 expression (Low, High). The total score of each patient was the sum of the points identified at the top of the scale for each factor and was then identified on the total points scale to determine the probability of 1-year, 3-year, and 5-year OS. **(B)** Plots depict the calibration of the model in terms of the agreement between predicted and observed 3- and 5-year OS. Model performance is shown by the plot, relative to the 45-degree line, which represents perfect prediction. OS, overall survival.

We developed a nomogram that integrates predictive factors such as age, cancer stage, T stage, N stage, and GFPT1 expression to quantitatively assess overall survival (OS) probabilities at 1, 3, and 5 years for BRCA patients (Figure 3A). Each patient’s covariates were assigned specific point values, with higher total scores corresponding to lower predicted survival rates. Calibration plots demonstrated that the nomogram’s predictions closely aligned with the ideal model (Figure 3B), indicating strong predictive accuracy.

### 3.4 Functional annotation of genes and pathway analysis

We performed differential expression analysis between the GFPT1-Low and GFPT1-High groups in the BRCA cohort, identifying 354 differentially expressed genes (DEGs). Among these, 111 genes were upregulated and 243 were downregulated in the GFPT1-High group compared to the GFPT1-Low group (Supplementary Table S1). To explore how GFPT1 influences clinical outcomes in BRCA patients, we conducted GO and KEGG pathway enrichment analyses using the clusterProfiler package.

**FIGURE 4:**
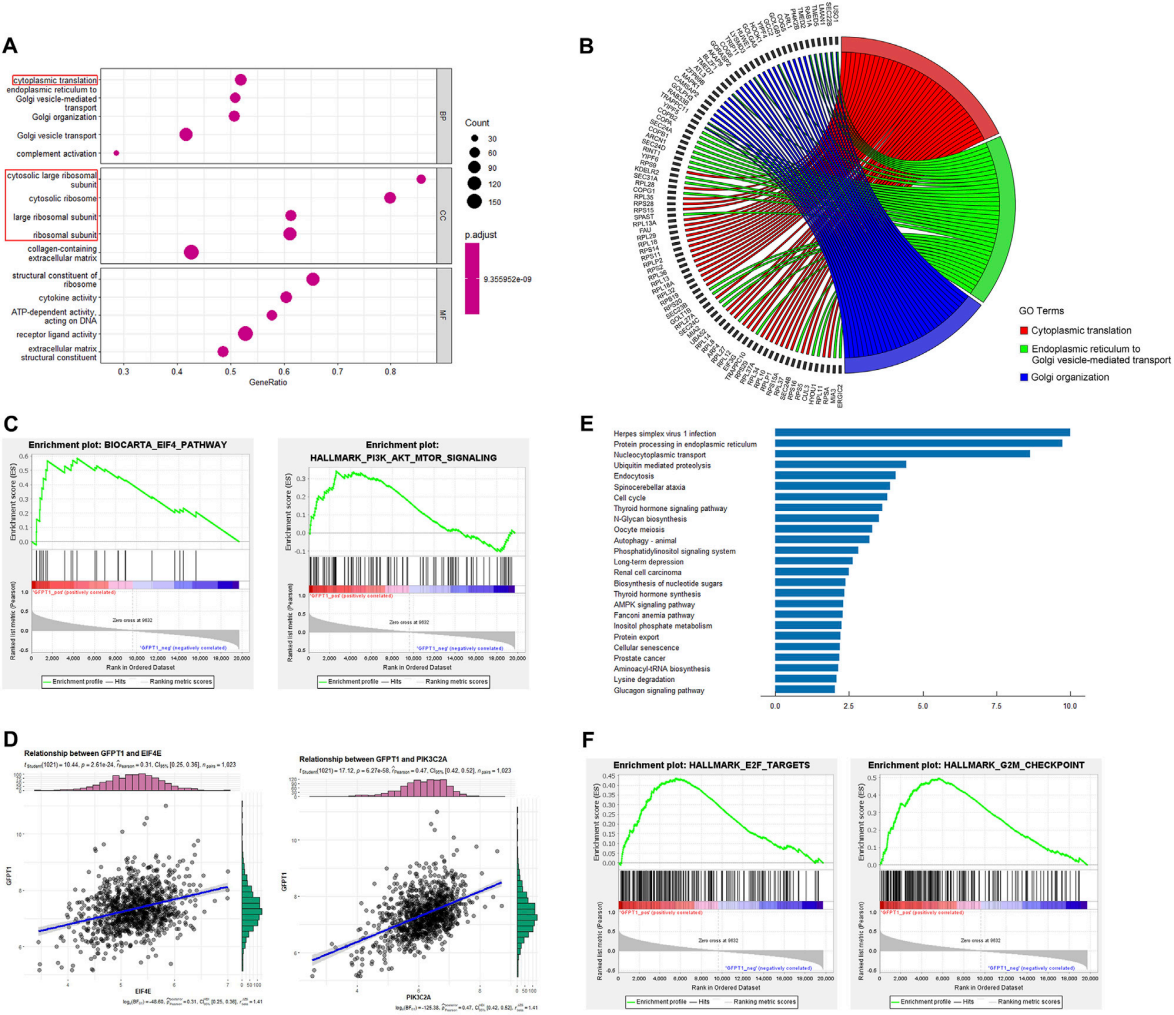
Functional enrichment analysis. **(A)** Top 5 (ranked by *p*-value) significantly enriched GO terms of three categories, including biological processes (BP), cellular components (CC) and molecular functions (MF). Gene ratio (*x*-axis) is the percentage of the number of genes present in this GO term over the total number of genes in this category, and the *y*-axis represents the significantly enriched GO terms. Ribosome-related GO terms are indicated by red box. **(B)** The top 3 BP GO terms and corresponding representative genes are described in detail by the correlation Circos plot. **(C)** GSEA for gene sets related with GFPT1 expression. The horizonal axis represents genes of BIOCARTA_EIF4_PATHWAY and HALLMARK_PI3K_AKT_MTOR_SIGNALING gene sets, ranked by decreasing risk score. The vertical axis represents enrichment score (ES). **(D)** Pearson correlation coefficient (r) and *p*-value (P) between EIF4E, PIK3C2A and GFPT1 gene expression. **(E)** Top 25 significantly enriched pathways in the KEGG pathway analysis. **(F)** GSEA for gene sets of HALLMARK_E2F_TARGETS and HALLMARK_G2M_CHECKPOINT gene sets, ranked by decreasing risk score. GO, Gene Ontology. GSEA, Gene Set Enrichment Analysis. KEGG, Kyoto Encyclopedia of Genes and Genomes.

As shown in Figure 4A, the GO enrichment analysis for biological processes (BP) revealed that the GFPT1-High group was enriched in processes such as “cytoplasmic translation,” “endoplasmic reticulum to Golgi vesicle-mediated transport,” “Golgi organization,” “Golgi vesicle transport,” and “complement activation.” The top three enriched BP GO terms and their associated genes were visualized using correlation Circos plots (Figure 4B). Additionally, the top four enriched cellular component (CC) GO terms were all related to ribosomes, including “cytosolic large ribosomal subunit,” “cytosolic ribosome,” “large ribosomal subunit,” and “ribosomal subunit.”

Gene Set Enrichment Analysis (GSEA) revealed significant associations between oncogenic signaling pathways regulating translation, such as the EIF4 pathway and HALLMARK_PI3K_AKT_MTOR_SIGNALING gene sets, and the GFPT1-High expression group (Figure 4C). Positive correlations were observed between GFPT1 and EIF4E (r = 0.31, *p* = 2.61E-24), as well as GFPT1 and PIK3C2A (r = 0.47, *p* = 6.27E-58) (Figure 4D). These findings suggest that tumors with higher GFPT1 expression are more likely to exhibit enhanced translational activity, promoting mRNA translation crucial for cancer progression.

Moreover, KEGG pathway enrichment analysis identified several oncogenic pathways enriched in the GFPT1-High group, including the cell cycle, autophagy, nucleotide sugar biosynthesis, AMPK signaling pathway, and Fanconi anemia pathway, involving genes linked to breast cancer susceptibility (Fang et al., 2020) (Figure 4E). GSEA further showed significant enrichment of cell cycle-related genes, such as HALLMARK_E2F_TARGETS and HALLMARK_G2M_CHECKPOINT, in the GFPT1-High group (Figure 4F). These results highlight the role of elevated GFPT1 expression in promoting cell cycle progression and driving cancer development.

### 3.5 Relationship between somatic mutations and GFPT1 expression

Previous studies have identified somatic mutations as critical drivers of cancer development (Oh and Sung, 2021). To investigate differences in somatic mutations between the GFPT1-High and GFPT1-Low groups in the BRCA cohort, we analyzed the TCGA-BRCA dataset using the Maftools package. The mutational landscape revealed that missense mutations were the most common, with single nucleotide polymorphisms (SNPs) being the predominant variant type in both groups. Among single nucleotide variants (SNVs), C > T transitions were the most frequently observed (Supplementary Figure S4A, B).

**FIGURE 5:**
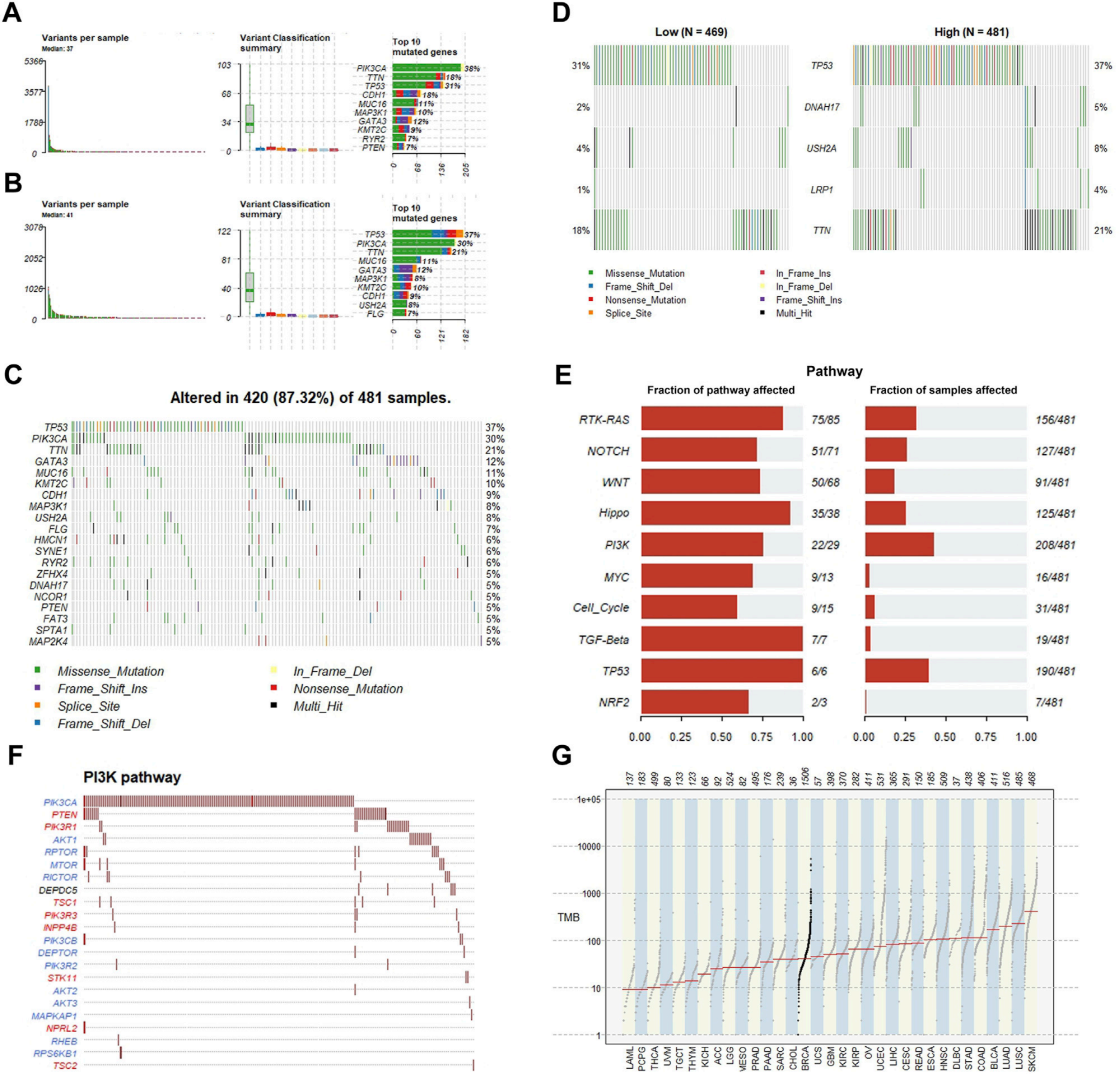
Association between GFPT1 and gene alterations in TCGA-BRCA cohort. **(A–B)** Summary of somatic mutations, including variant per sample, variant classification summary and top 10 mutated genes in BC patients. GFPT1-Low **(A)** and GFPT1-High **(B)** groups are shown respectively. **(C)** Common tumor-related gene mutation information illustrated in the somatic mutation spectrum in GFPT1-High groups in BRCA. Somatic landscape and the genes in the top 20 of the population mutation frequency are shown in the figure. Genes are sorted according to their mutation frequency. **(D)** Mutation landscape of representative mutated genes between GFPT1-Low and GFPT1-High BC groups. **(E)** Fractions of pathways involving mutated genes in GFPT1-High BC groups. **(F)** Maftools pathway analysis of mutated genes in PI3K pathway. **(G)** Comparison of mutation burden of GFPT1-High BRCA pilot cohort across TCGA datasets. Samples of GFPT1-High BRCA cohort are indicated by black. TMB, tumor mutation burden.

Compared to the GFPT1-Low group, the GFPT1-High group exhibited a higher median mutational burden per sample (median: 41), with TP53 mutations present in 37% of samples—a significantly higher proportion than in the GFPT1-Low group (Figures 5A, B). The Oncoplot function visualized the top 20 mutated genes in both groups, displayed in waterfall plots for the GFPT1-High (Figure 5C) and GFPT1-Low groups (Supplementary Figure S5C). Notably, genes such as TP53, DNAH17, USH2A, LRP1, and TTN were more frequently mutated in the GFPT1-High group compared to the GFPT1-Low group (Figure 5D).

Pathway analysis revealed that oncogenic pathways, particularly the PI3K pathway, were significantly enriched among the mutated genes in the GFPT1-High group (Figure 5E). Figure 5F highlights the specific mutated genes within the PI3K pathway. Additionally, when comparing mutation rates between the GFPT1-High BRCA cohort and tumor mutation burdens (TMBs) across various cancer types in the TCGA dataset, BRCA exhibited a median mutation rate, ranking between cholangiocarcinoma and uterine carcinosarcoma (Figure 5G).

### 3.6 Relevance of GFPT1 expression to immune infiltration

**FIGURE 6:**
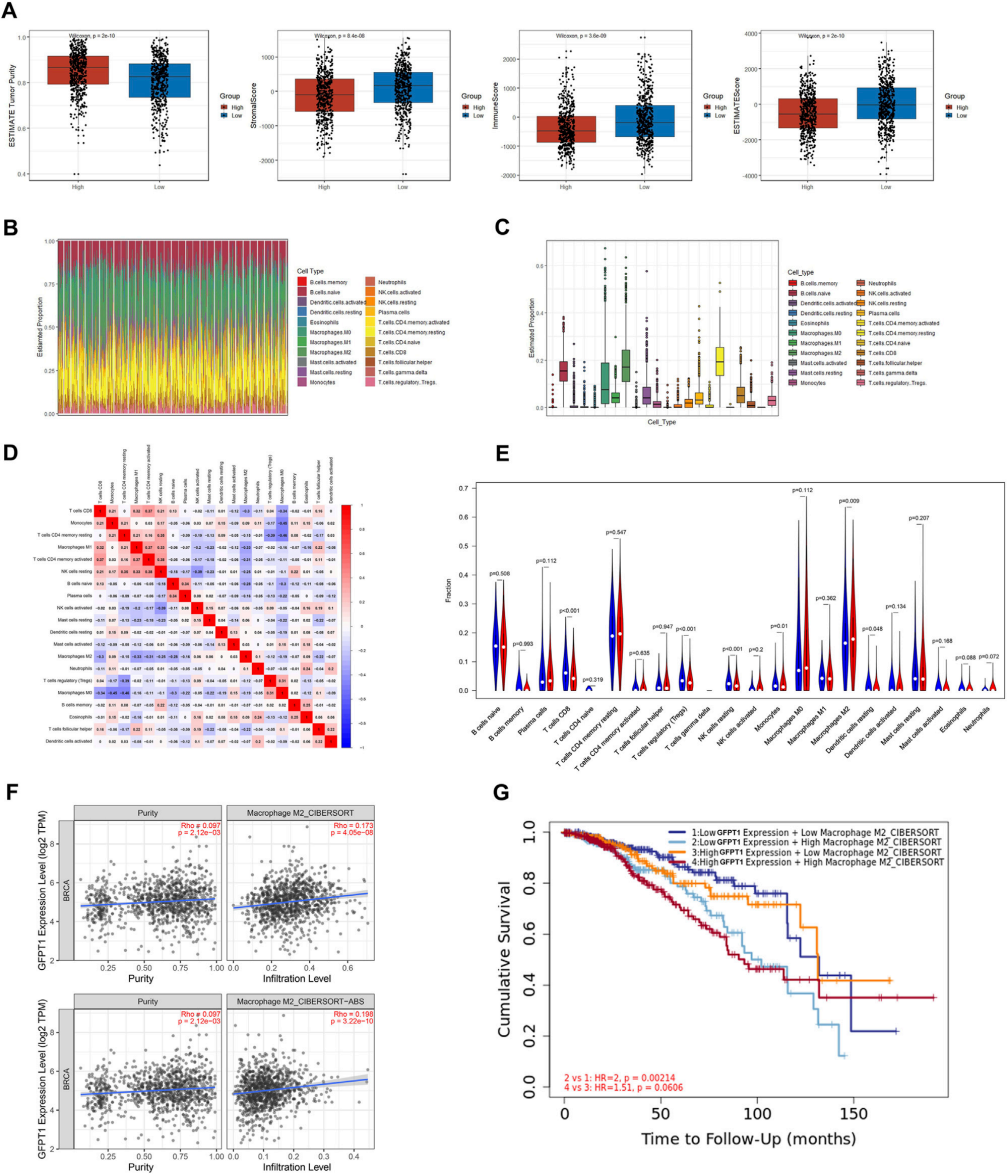
The correlation of TIICs with GFPT1 expression in BRCA. **(A)** Comparison of tumor purity, stromal score, immune score, and ESTIMATE score between GFPT1-Low and GFPT1-High groups according to the ESTIMATE tool. **(B–C)** Distribution and relative abundance of immune cell type fractions in BRCA. **(D)** Correlation matrix of TIICs proportions. **(E)** Violin plot comparing the proportions of TIICs between GFPT1-Low and GFPT1-High expression BRCA samples, respectively. Blue, GFPT1-Low groups. Red, GFPT1-High groups. **(F)** TIMER2.0 analysis of the correlation between GFPT1 expression and M2 macrophages infiltration in BRCA. **(G)** Outcome module of TIMER2.0 exploring the association between M2 macrophages infiltrates, GFPT1 expression, and clinical outcome in BRCA. The hazard ratio and the log-rank *p*-value for KM curve is shown on the KM curve plot. TIICs, tumor-infiltrating immune cells.

Tumors are known to elicit immune responses by presenting antigens, which lead to infiltration of lymphocytes into both the tumor and its surrounding stroma (Rohan et al., 2021). Previous studies have shown that breast cancer tissues exhibit a suppression of the adaptive immune system and an activation of the innate immune system (DeNardo and Coussens, 2007). To explore the immune landscape in breast cancer, we used the ESTIMATE algorithm to assess immune scores, stromal scores, and tumor purity between the GFPT1-Low and GFPT1-High groups in BRCA (Newman et al., 2015). Our analysis revealed that the GFPT1-High group exhibited significantly higher tumor purity, along with markedly lower stromal, immune, and ESTIMATE scores, suggesting reduced immune cell infiltration in this group. This immunosuppressive environment likely supports tumor progression (Figure 6A). Figures 6B,C highlight the distribution and percentages of immune cells in the GFPT1-High group, showing that macrophages and T lymphocytes were the dominant immune cells infiltrating the breast cancer tissues. Figure 6D illustrates weak to moderate correlations between representative immune cells infiltrating the tumor microenvironment in the GFPT1-High BRCA group.

To further investigate the differences in tumor-infiltrating immune cells (TIICs) between the GFPT1-Low and GFPT1-High groups, we applied the CIBERSORT algorithm to breast cancer patients from the TCGA cohort. The violin plot revealed significant reductions in CD8^+^ T cells (*p* < 0.001), Tregs (*p* < 0.001), resting NK cells (*p* < 0.001), monocytes (*p* = 0.01), and resting dendritic cells (*p* = 0.048) in the GFPT1-High group (Figure 6E). Research has shown that M2 macrophage polarization is strongly associated with breast cancer aggressiveness, larger tumor size, advanced stages, and angiogenesis (Jayasingam et al., 2019; Jeong et al., 2019). In our study, elevated GFPT1 expression was linked to a significantly increased proportion of M2 macrophages, suggesting a direct correlation between GFPT1 expression and the anti-inflammatory infiltration of M2 macrophages in breast cancer tissues (Figure 6E).

Further analysis using TIMER2.0 demonstrated a robust association between GFPT1 expression and M2 macrophage infiltration in breast cancer tissues (Figure 6F). Additionally, TIMER2.0 revealed that patients with low GFPT1 expression and low M2 macrophage infiltration had the most favorable prognosis, while those with high GFPT1 expression and high M2 macrophage infiltration had the poorest survival outcomes (Figure 6G). These findings underscore the critical role of GFPT1 expression, M2 macrophage infiltration, and their impact on clinical outcomes in BRCA patients.

### 3.7 Drug sensitivity prediction based on GFPT1 expression

**FIGURE 7:**
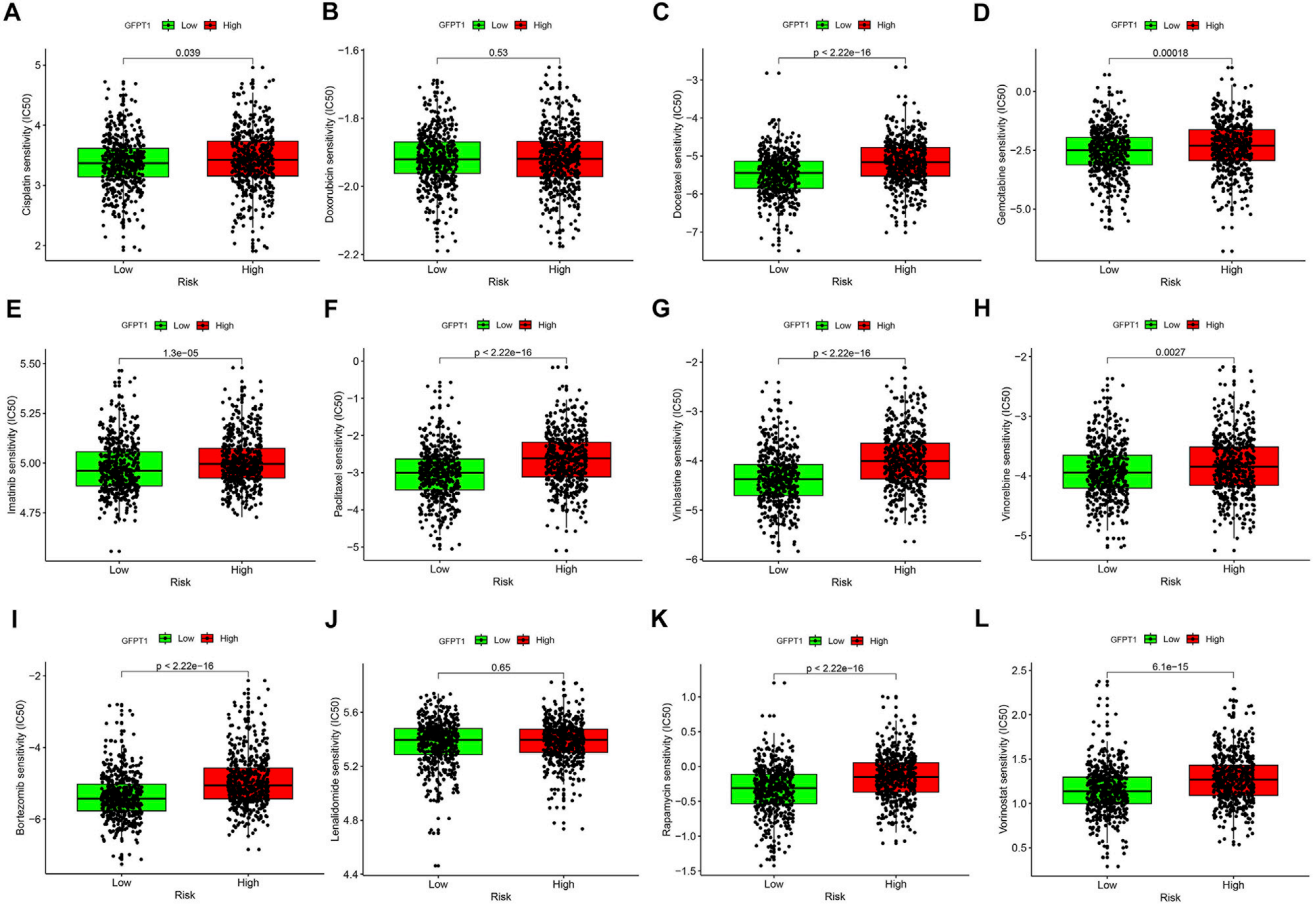
Drug sensitivity prediction using the IC50 values based on GFPT1 expression in TCGA-BRCA. (A–L) The IC50 values for BC-concerning chemotherapeutics acquired from pRRophetic algorithm were compared between GFPT1-Low and GFPT1-High groups from TCGA-BRCA cohort. The IC50 values of 8 cytotoxic chemotherapeutics Cisplatin **(A)**, Doxorubicin **(B)**, Docetaxel **(C)**, Gemcitabine **(D)**, Imatinib **(E)**, Paclitaxel **(F)**, Vinblastine **(G)**, Vinorelbine **(H)**, Bortezomib **(I)**, Lenalidomide **(J)**, Rapamycin **(K)**, and Vorinostat **(L)** were compared respectively. BC, breast cancer. (Mann-Whitney *U* test, *p < 0.05; **p < 0.01; and ***p < 0.001).

To assess the clinical significance of GFPT1 expression, we applied the pRRophetic algorithm and package to analyze the half-maximal inhibitory concentration (IC50) values of eight commonly used chemotherapeutic agents in breast cancer treatment, including Cisplatin, Doxorubicin, Docetaxel, Gemcitabine, Imatinib, Paclitaxel, Vinblastine, and Vinorelbine, across GFPT1-High and GFPT1-Low groups in BRCA. The results demonstrated significantly lower IC50 values in the GFPT1-Low group for most drugs: Cisplatin (*p* = 0.039), Docetaxel (*p* = 2.22E-16), Gemcitabine (*p* = 1.8E-04), Imatinib (*p* = 1.3E-05), Paclitaxel (*p* = 2.22E-16), Vinblastine (*p* = 2.22E-16), and Vinorelbine (*p* = 2.7E-03), with the exception of Doxorubicin (*p* = 0.530) (Figures 7A–H).

We further evaluated the IC50 values of immunomodulatory drugs, including Bortezomib (Figure 7I), Lenalidomide (Figure 7J), Rapamycin (Figure 7K), and Vorinostat (Figure 7L), based on GFPT1 expression. The data revealed that GFPT1 could serve as a potential biomarker for predicting the efficacy of immune-related drugs such as Bortezomib (*p* = 2.22E-16), Rapamycin (*p* = 2.22E-16), and Vorinostat (*p* = 6.1E-15). These findings highlight the potential of GFPT1 expression levels as a valuable predictor of chemotherapy response and immunotherapy outcomes in invasive breast carcinoma.

### 3.8 Identification of GFPT1 protein expression in breast cancer cell lines and its influence on breast cancer cell proliferation

**FIGURE 8:**
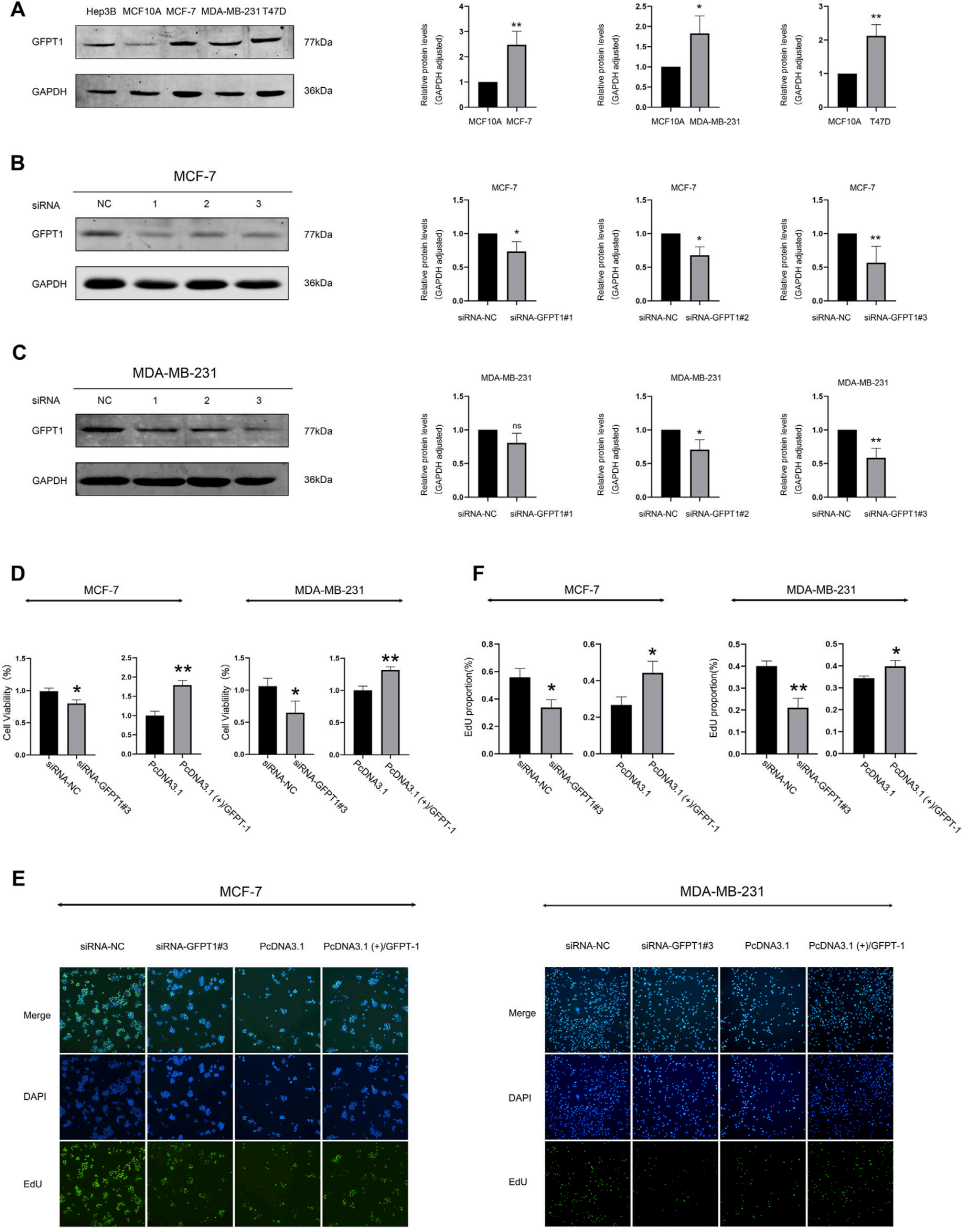
Protein expression of GFPT1 and effect of GFPT1 gene knockdown on the proliferation ability of breast cancer cells. **(A)** Western blot analysis of endogenous GFPT1 protein levels in Hep3B2.1-7, MCF10A, MCF-7, MB-MDA-231, and T47D cells (n = 3, mean ± SD; *p < 0.05 vs. MCF10A cell control, **p < 0.01 vs. MCF10A cell control). **(B–C)** Western blot displayed MCF-7 **(B)** and MDA-MB-231 **(C)** cells transfected for 48 h with siRNA-NC, siRNA-GFPT1#1, siRNA-GFPT1#2, and siRNA-GFPT1#3 (75 nM, 50 nM) (n = 3, mean ± SD, *p < 0.05 vs. siRNA-NC control, **p < 0.01 vs. siRNA-NC control). **(D)** Cell viability in MCF-7 and MDA-MB-231 cells was assessed after transfection for 48 h with siRNA-NC, siRNA-GFPT1#3, PcDNA3.1, and PcDNA3.1 (+)/GFPT1. **(E)** Representative EdU cell proliferation assay profiles in MCF-7 and MDA-MB-231 cells after transfection with siRNA-NC, siRNA-GFPT1#3, PcDNA3.1, and PcDNA3.1 (+)/GFPT1 (×100 magnification). **(F)** Quantification of EdU-positive cells following transfection with siRNA-NC, siRNA-GFPT1#3, PcDNA3.1, and PcDNA3.1 (+)/GFPT1 in MCF-7 and MDA-MB-231 cells.

We next assessed GFPT1 protein levels and compared its expression in MCF10A cells, derived from normal human mammary epithelial cells, with breast cancer cell lines MCF-7, MB-MDA-231, and T47D. Our results demonstrated a decrease in GFPT1 protein expression in the breast carcinoma cell lines (MCF-7, MB-MDA-231, and T47D) compared to the MCF-10A cell line (Figure 8A). Hep3B2.1-7 cells, a human hepatocellular carcinoma cell line, were used as a positive control for GFPT1 protein expression.

To investigate the role of GFPT1 in breast cancer cell growth, we first employed RNA interference to suppress GFPT1 expression in MCF-7 and MDA-MB-231 cells. Western blot analysis confirmed the successful downregulation of GFPT1 protein levels by GFPT1 siRNA, with siRNA-GFPT1#3 showing the most significant reduction in both cell lines (Figures 8B, C). Subsequent analysis of cell viability following GFPT1 inhibition revealed a marked decrease in survival rates in both MCF-7 and MDA-MB-231 cells transfected with siRNA-GFPT1#2 and siRNA-GFPT1#3 (Supplementary Figure S5A, B). To further explore the function of GFPT1, we overexpressed GFPT1 by transfecting MCF-7 and MDA-MB-231 cells with PcDNA3.1 (+)/GFPT1 plasmids, with overexpression efficiency confirmed via western blot (Supplementary Figure S6A, B). Overexpression of GFPT1 significantly increased cell viability in both breast cancer cell lines (Figure 8D).

Additionally, we assessed cell proliferation using the EdU staining assay. The results demonstrated that GFPT1 knockdown with siRNA-GFPT1#3 significantly inhibited cell proliferation, while overexpression of GFPT1 enhanced proliferation in both MCF-7 and MDA-MB-231 cells (Figures 8E, F). These findings suggest that GFPT1 is critical for the growth and proliferation of breast cancer cells *in vitro*.

### 3.9 Impact of GFPT1 expression on apoptosis and migration in breast cells

**FIGURE 9:**
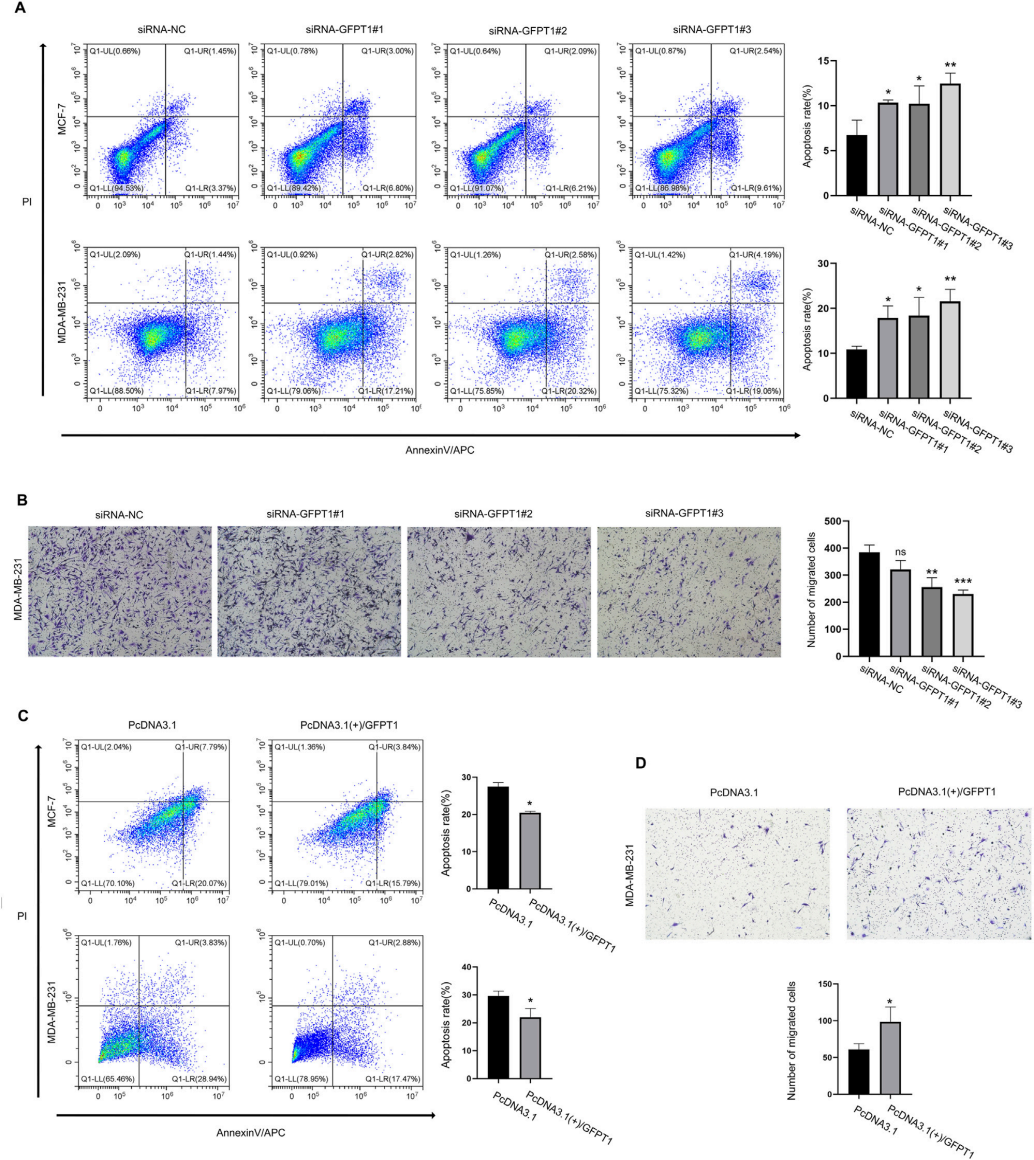
Knockdown of GFPT1 promoted apoptosis and inhibited migration of breast cancer cells. **(A)** Apoptosis in MCF-7 and MDA-MB-231 cells was assessed after transfection for 48 h with siRNA-NC, siRNA-GFPT1#1, siRNA-GFPT1#2, and siRNA-GFPT1#3 (75 nM, 50 nM) (n = 3 in biological and experimental triplicate, one-way ANOVA followed by Tukey’s *post hoc* test for multiple groups comparison and t-test between columns, *p < 0.05 vs. siRNA-NC control, **p < 0.01 vs. siRNA-NC control). **(B)** Migration in MDA-MB-231 cells was examined after transfection for 48 h with siRNA-NC, siRNA-GFPT1#1, siRNA-GFPT1#2, and siRNA-GFPT1#3 (50 nM). Scale bars represent 100 μm (n = 3 in biological and experimental triplicate, one-way ANOVA followed by Tukey’s *post hoc* test for multiple groups comparison and t-test between columns, ns *p* > 0.05 vs. siRNA-NC control, **p < 0.01 vs. siRNA-NC control, ***p < 0.001 vs. siRNA-NC control). ns, not significant. **(C)** Apoptosis in MCF-7 and MDA-MB-231 cells was assessed after transfection for 48 h with PcDNA3.1 or PcDNA3.1 (+)/GFPT1 plasmids. **(D)** Migration in MDA-MB-231 cells was examined after transfection for 48 h with PcDNA3.1, and PcDNA3.1 (+)/GFPT1.

To investigate the influence of GFPT1 on apoptosis in breast cancer cells, we employed APC/Annexin V and PI dual-staining flow cytometry to analyze apoptosis and necrosis in human MCF-7 and MDA-MB-231 cells. This analysis included early apoptotic (APC+/PI-) and late apoptotic/necrotic cells (APC+/PI+). As shown in Figure 9A, the percentages of apoptotic MCF-7 cells significantly increased with various GFPT1 siRNAs: siRNA-GFPT1#1 resulted in approximately 6.80% early apoptosis and 3.00% late apoptosis/necrosis; siRNA-GFPT1#2 showed around 6.21% early apoptosis and 2.09% late apoptosis/necrosis; and siRNA-GFPT1#3 led to approximately 9.61% early apoptosis and 2.54% late apoptosis/necrosis. Similar trends were observed in MDA-MB-231 cells, where siRNA-GFPT1#1 induced about 17.21% early apoptosis and 2.82% late apoptosis/necrosis; siRNA-GFPT1#2 resulted in approximately 20.32% early apoptosis and 2.58% late apoptosis/necrosis; and siRNA-GFPT1#3 showed around 19.06% early apoptosis and 4.19% late apoptosis/necrosis following GFPT1 siRNA transfection (Figure 9A). Overall, these results suggest that suppressing GFPT1 expression promotes apoptosis and necrosis, thereby hindering the growth of breast cancer cells. We also investigated the impact of GFPT1 overexpression on apoptosis in breast cancer. As illustrated in Figure 9C, the proportion of early and late apoptotic cells was significantly reduced in MCF-7 and MDA-MB-231 cells overexpressing GFPT1 compared to mock control cells. These results clearly indicate that GFPT1 overexpression attenuates apoptotic cell death in both MCF-7 and MDA-MB-231 cell lines.

We also investigated the potential effects of GFPT1 on other types of cell death. The expression of GSDMD, a key effector of pyroptosis, was evaluated in MCF-7 and MDA-MB-231 breast cancer cell lines following transfection with GFPT1-targeting siRNA (siRNA-GFPT1#3), an empty vector control (PcDNA3.1), and a GFPT1 overexpression vector (PcDNA3.1 (+)/GFPT1). However, we did not observe any significant differences in the expression of cleaved GSDMD protein, the active form associated with pyroptosis, among the various treatment groups (Supplementary Figure S7A, B). These findings indicate that GFPT1 does not play a regulatory role in modulating pyroptosis in breast cancer cells.

Given that tumor metastasis involves the migration and invasion of cancer cells, we conducted a transwell migration assay to determine the impact of GFPT1 siRNA and GFPT1 overexpression on breast cancer cell migration. The findings indicated that reducing GFPT1 expression significantly impaired the migration ability of MDA-MB-231 cells while the migration ability of the cells was significantly increased after GFPT1 overexpression, suggesting that GFPT1 plays a role in promoting breast cancer cell migration and metastasis (Figures 9B, D).

## 4 Discussion

Metabolic reprogramming is a fundamental trait of cancer cells that distinguishes them from normal cells. Cancer cells prefer utilizing glycolysis over mitochondrial oxidative phosphorylation to generate energy, a phenomenon commonly referred to as the Warburg effect (Shin and Koo, 2021; Yu et al., 2024). The hexosamine biosynthesis pathway (HBP) is a branch of glucose metabolism that integrates carbohydrates (glucose), lipids (acetyl-CoA), amino acids (glutamine), and nucleotides (UTP) to produce UDP-GlcNAc (Vasconcelos-Dos-Santos et al., 2018). UDP-GlcNAc acts as a sugar donor and generates activated monosaccharides like UDP-GalNAc and CMP-Neu5Ac, essential for glycosylation reactions (Vasconcelos-Dos-Santos et al., 2015). Dysregulated glycosylation is a recognized hallmark of cancer, contributing to tumorigenesis and cancer progression (de Queiroz et al., 2019).

Breast cancer remains one of the leading causes of cancer-related mortality worldwide, with more than two million new cases diagnosed annually. While early-stage breast cancer is often curable, metastatic breast cancer (MBC), characterized by its spread to distant organs, poses a significant clinical challenge due to its aggressive nature and resistance to conventional therapies (Hattori and Iwata, 2018). Among the subtypes, triple-negative breast cancer (TNBC) is associated with particularly poor outcomes due to its high recurrence rate and lack of targeted therapies. Understanding the molecular drivers behind breast cancer metastasis and therapy resistance is critical to improving patient outcomes. Our study highlights the clinical relevance of GFPT1, the rate-limiting enzyme in the HBP, in breast cancer. Elevated GFPT1 expression has been implicated in tumor growth, angiogenesis, metastasis, and resistance to chemotherapy (Akella et al., 2019). While previous studies have pointed to GFPT1’s involvement in TNBC and its association with poor prognosis (Dong et al., 2016), our findings extend its significance across invasive breast cancer subtypes.

We observed that GFPT1 expression is significantly elevated in breast cancer tissues compared to normal tissues, with a notable correlation between high GFPT1 levels and advanced tumor stages, highlighting its potential role in driving breast cancer progression. Importantly, high GFPT1 expression was linked to worse overall survival (OS) rates in both the TCGA-BRCA cohort and independent GEO datasets, establishing GFPT1 as an independent prognostic factor for breast cancer. The predictive nomogram model we constructed further underscores the potential utility of GFPT1 as a clinical tool to predict patient outcomes.

In terms of clinical significance, our findings suggest that GFPT1 may serve as both a prognostic biomarker and a therapeutic target in breast cancer. The positive association between GFPT1 and tumor progression, combined with its role in chemotherapy resistance, suggests that targeting GFPT1 could enhance the efficacy of current treatment strategies, particularly in patients with aggressive or advanced-stage disease. The strong correlation we observed between GFPT1 expression and immune cell infiltration, particularly with anti-inflammatory M2 macrophages, may also shed light on how GFPT1 influences the tumor microenvironment, potentially contributing to immune evasion and resistance to immune checkpoint inhibitors. Our *in vitro* experiments further emphasize the clinical importance of GFPT1, as silencing its expression significantly impaired breast cancer cell viability, migration, and survival, while GFPT1 overexpression enhanced these properties. This regulatory role of GFPT1 in modulating cell viability and apoptosis suggests that targeting GFPT1 could be a promising therapeutic strategy, particularly in metastatic or chemotherapy-resistant breast cancer. Moreover, bioinformatics analyses revealing GFPT1’s involvement in key oncogenic pathways, including autophagy, nucleotide sugar biosynthesis, and cell cycle regulation, highlight its function as a central regulator of metabolic and proliferative processes in breast cancer cells.

Despite the promising implications of our findings, the study has limitations. Most notably, the reliance on bioinformatic analysis using the TCGA database without clinical sample validation limits the direct translational applicability of our results. Future research should focus on validating these findings in clinical settings and elucidating the precise mechanisms by which GFPT1 regulates oncogenic pathways, immune cell infiltration, and chemotherapy resistance in breast cancer.

In conclusion, our study underscores the critical role of GFPT1 in breast cancer progression and metastasis. The association between GFPT1 expression and poor patient outcomes, as well as its influence on immune infiltration and chemotherapy sensitivity, suggests that GFPT1 could serve as a novel therapeutic target. Targeting GFPT1 may offer new avenues for the treatment of breast cancer, particularly in cases of advanced disease or chemoresistance.

## 5 Conclusion

In summary, our research is the first to identify GFPT1 as a prognostic marker in breast cancer. Silencing GFPT1 in breast cancer cells reduced viability, induced apoptosis and necrosis, and inhibited migration. Additionally, GFPT1 was found to correlate with M2 macrophage infiltration and predict chemotherapy response in invasive breast cancer.

## Data Availability

The original contributions presented in the study are included in the article/Supplementary Material, further inquiries can be directed to the corresponding authors.
