# Supplementary material for: Bioinformatics insights into the role of GFPT1 in breast invasive carcinoma: implications for tumor prognosis, immune modulation, and therapeutic applications
Source: Front Genet. 2024 Nov 22;15:1482929. doi: 10.3389/fgene.2024.1482929 (PMC11633769; doi:10.3389/fgene.2024.1482929)
Supplement: Supplementary file 1 [file DataSheet1.docx]

Supplementary Material

# Supplementary Figures and Tables

## Supplementary Figures


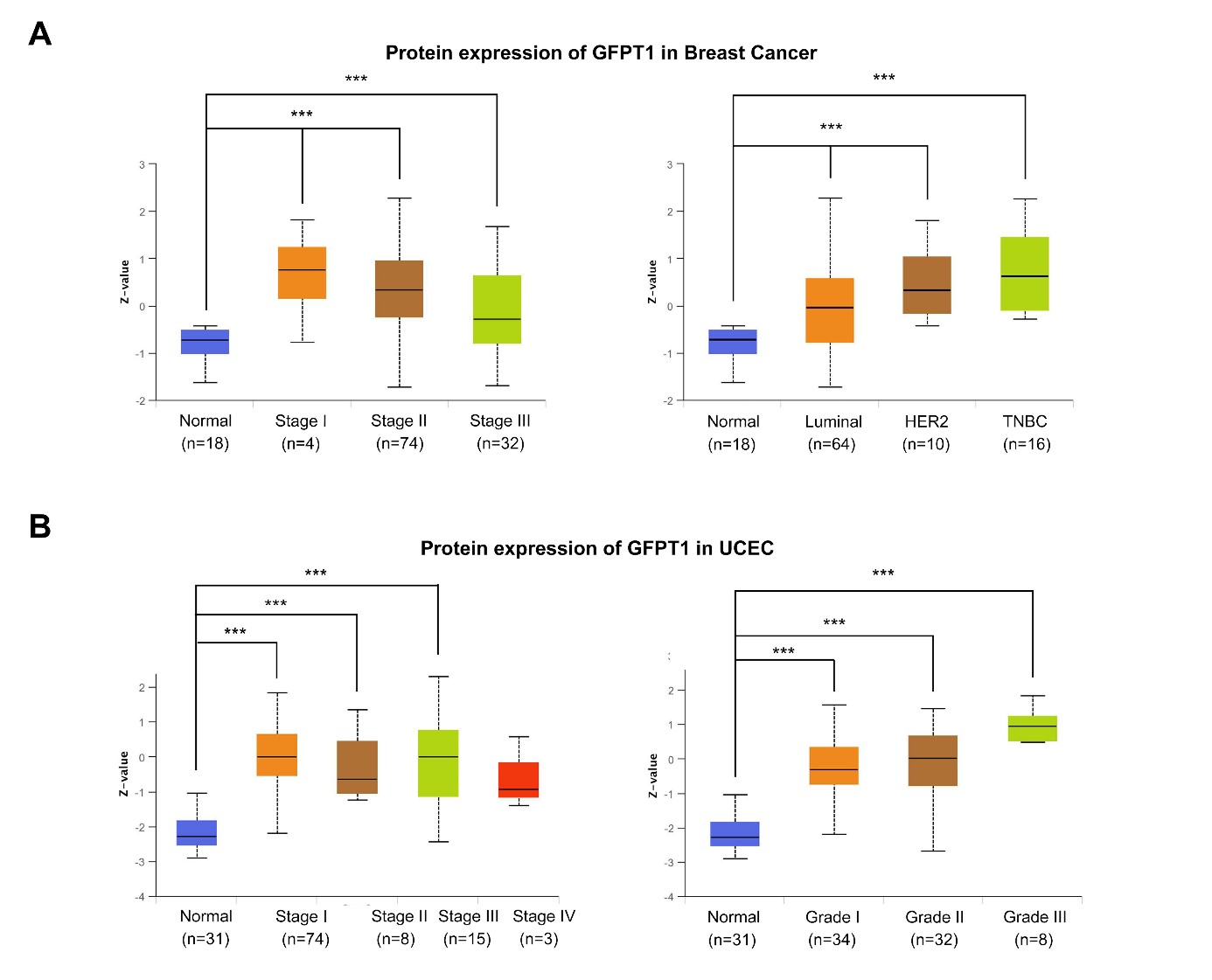


**Supplementary Figure 1.** GFPT1 proteomic expression in Breast Cancer and UCEC from CPTAC database. (A) The expression level of GFPT1 total protein was analyzed between normal tissues and tumor tissues from Breast Cancer by the main pathological stages (stage I, stage II, stage III) and by the major subclasses (Luminal, HER2 Positive, TNBC) based on the CPTAC dataset. (B) The expression level of GFPT1 total protein was analyzed between normal tissues and tumor tissues from UCEC by the main pathological stages (Stage I, Stage II, Stage III, Stage IV) and by the tumor grade (Grade I, Grade II, Grade III) based on the CPTAC dataset. Z-values represent standard deviations from the median across samples. *, p<0.05; **, p<0.01; ***, p<0.001.


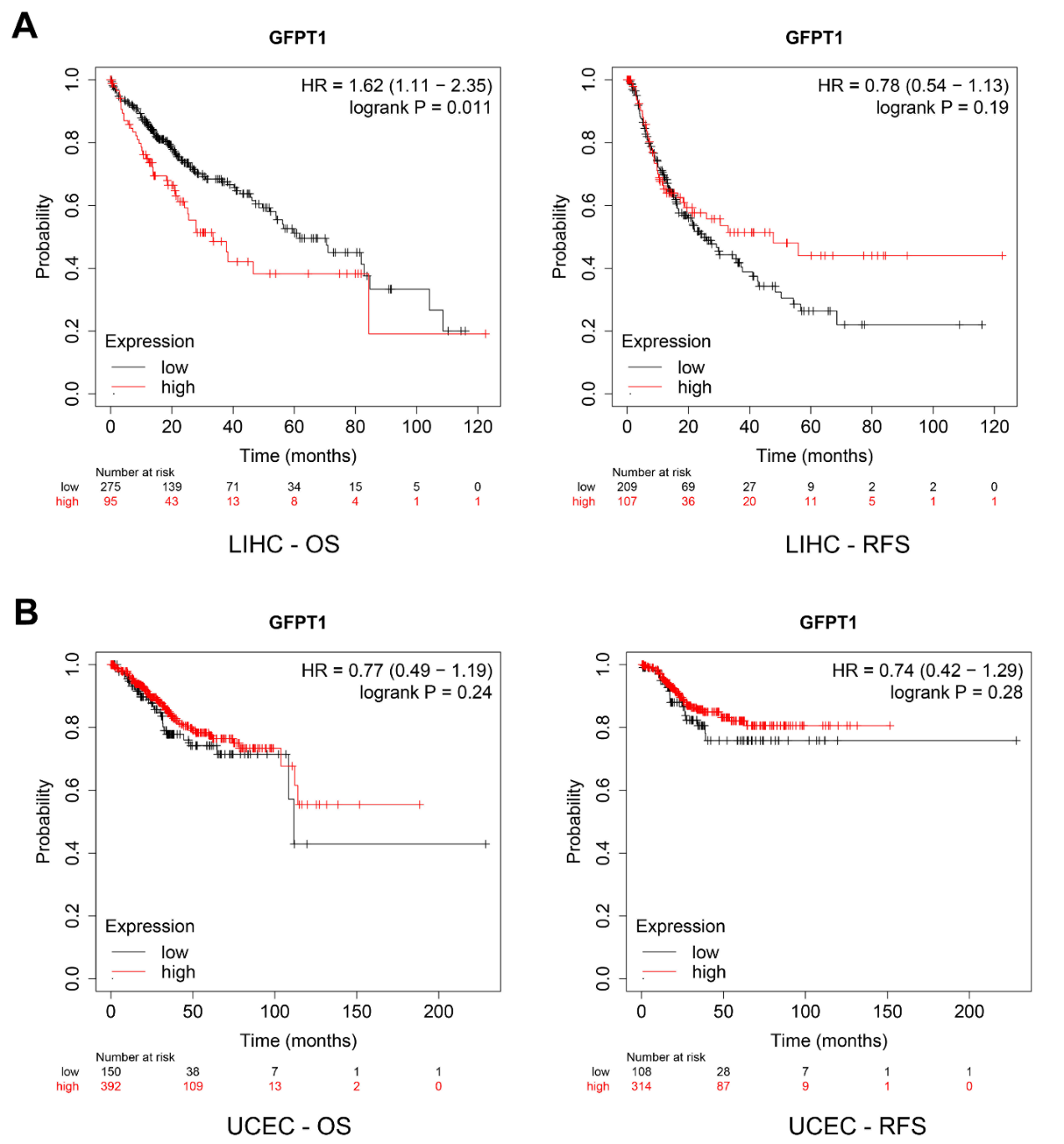


**Supplementary Figure 2.** Kaplan-Meier survival analysis for Overall survival (OS) and Progression-free survival (PFS) of LIHC and UCEC patients according to the GFPT1 expression. (A-B) The association of GFPT1 expression with Overall survival (OS) and Progression-free survival (PFS) was examined by Kaplan-Meier analysis in LIHC patients (A) and UCEC patients (B) respectively using the online survival analysis software (https://kmplot.com/analysis/).


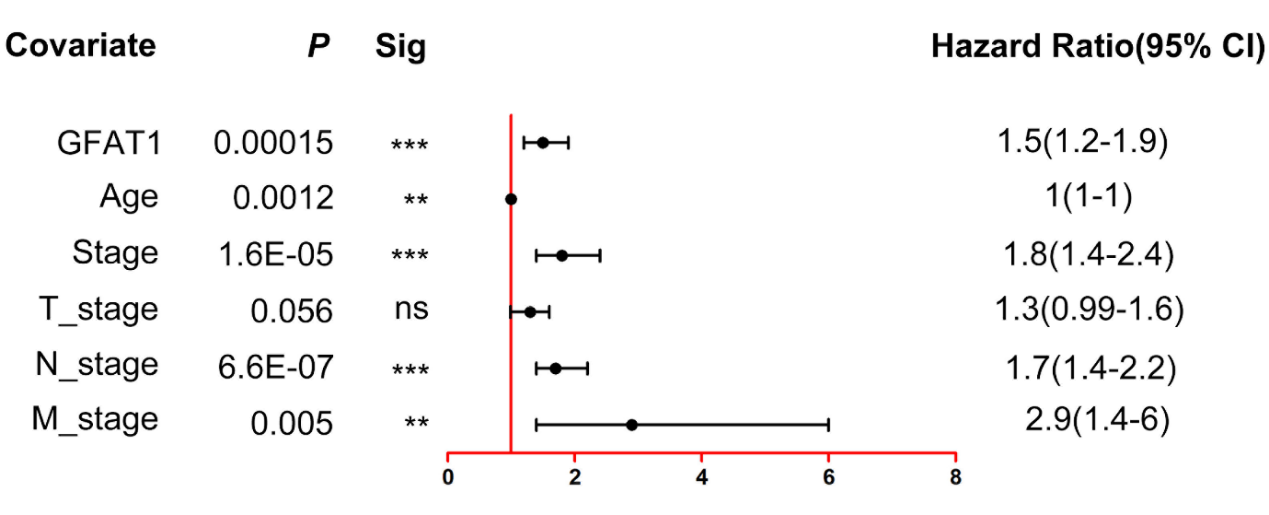


**Supplementary Figure 3.** Univariate Cox regression analysis of clinicopathological characteristics influencing the overall survival of breast cancer patients.


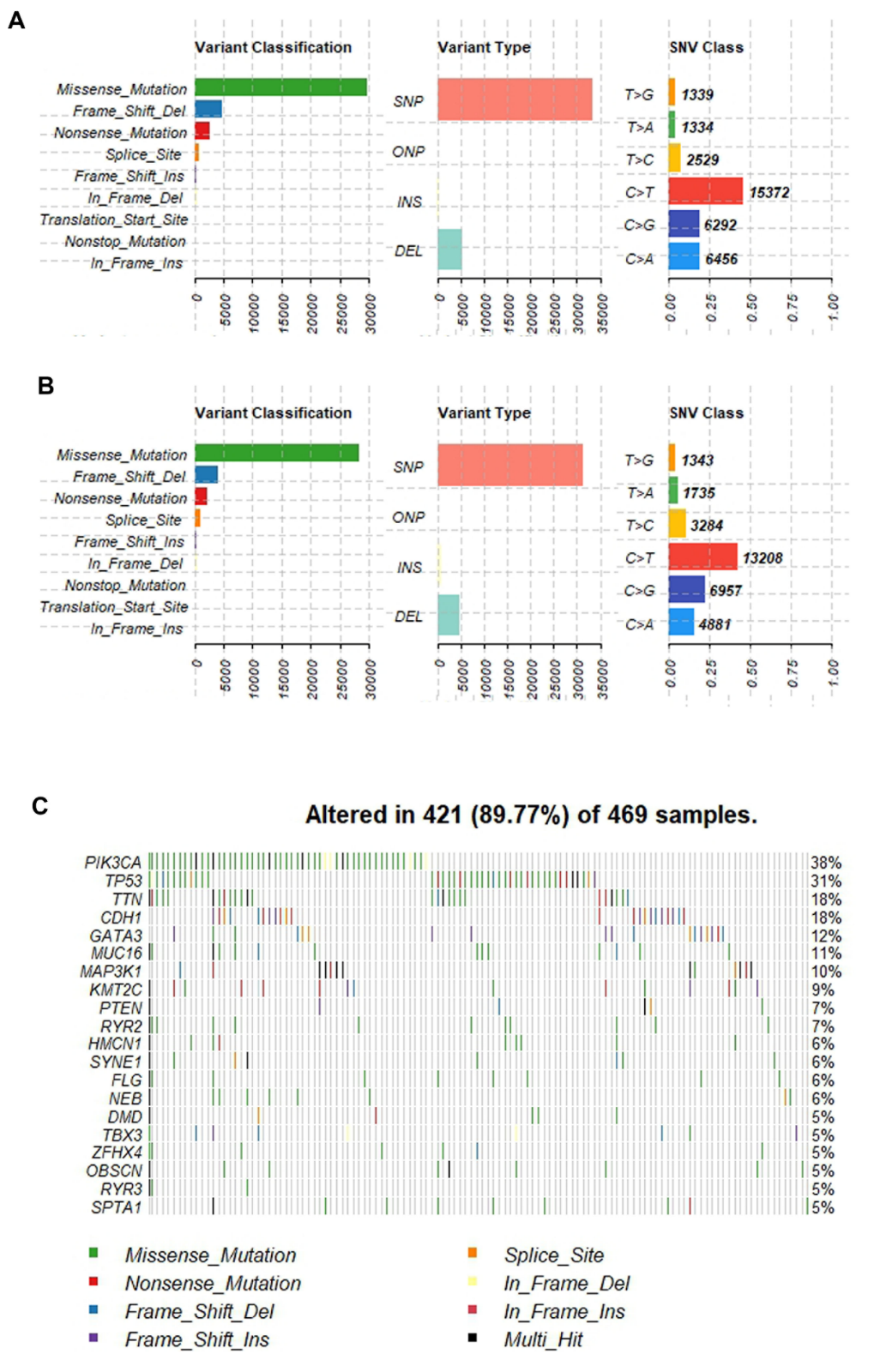


**Supplementary Figure 4.** Summary of somatic mutations between GFPT1-Low and GFPT1-Low groups and mutation landscape variation in GFPT1-Low groups. (A-B) Summary of somatic mutations, in-cluding the variant classification, variant type, SNV class in BC patients. GFPT1-Low (A) and GFPT1-High (B) groups are shown respectively. (C) Common tumor-related gene mutation information illustrated in the somatic mutation spectrum in GFPT1-Low groups in TCGA-BRCA. The genes in the top 20 of the population mutation frequency are shown in the figure.


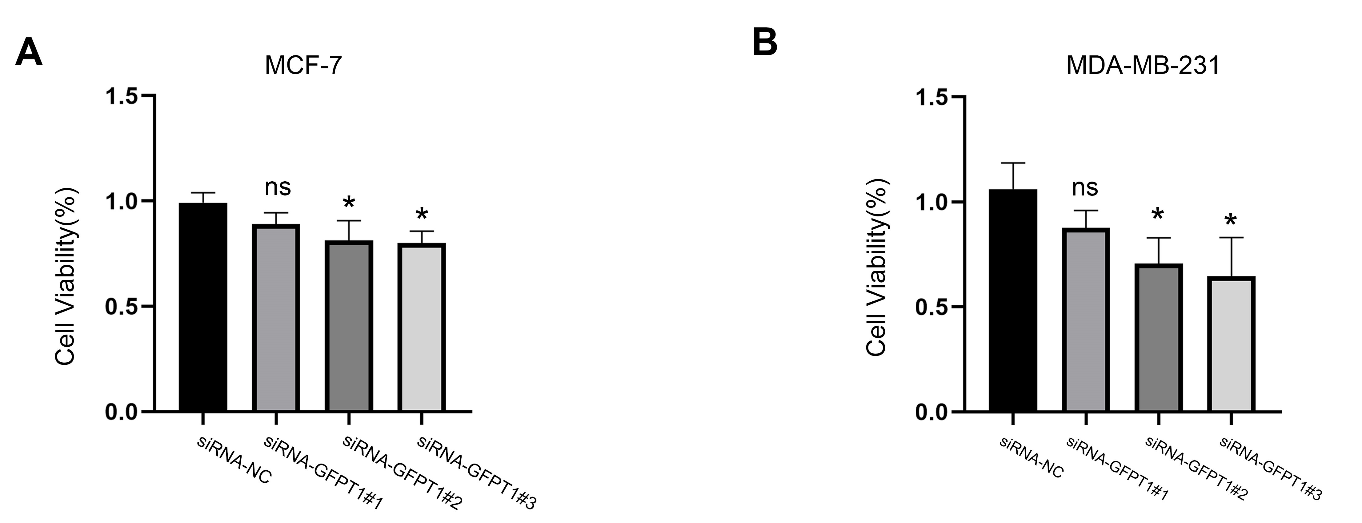


**Supplementary Figure 5.** (A-B) Cell viability in MCF-7 (A) and MDA-MB-231 (B) cells was assessed after transfection for 48 h with siRNA-NC, siRNA-GFPT1#1, siRNA-GFPT1#2, and siRNA-GFPT1#3 (75nM, 50nM) (n = 3 in biological and experimental triplicate, one-way ANOVA followed by Tukey’s post hoc test for multiple groups comparison and t-test between columns, nsp >0.05 vs. siRNA-NC control, * p < 0.05 vs. siRNA-NC control, ** p < 0.01 vs. siRNA-NC control). ns, not significant.


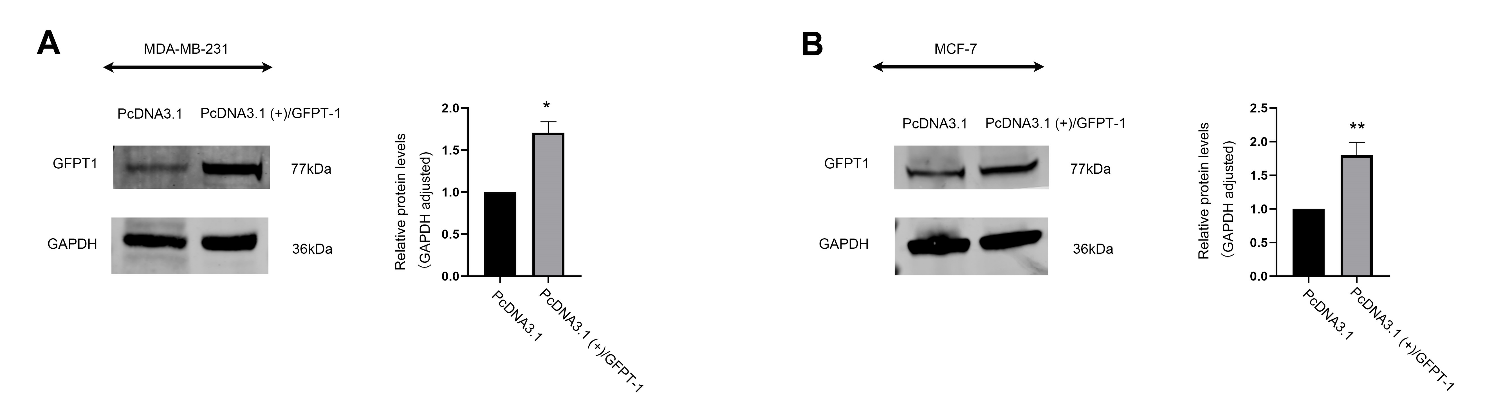


**Supplementary Figure 6.** Western blot analysis showed GFPT1 protein expression in MDA-MB-231 (A) and MCF-7 (B) cells after 48 hours of transfection with PcDNA3.1 or PcDNA3.1(+)/GFPT1 plasmids (n=3, mean±SD, *p < 0.05, **p < 0.01).


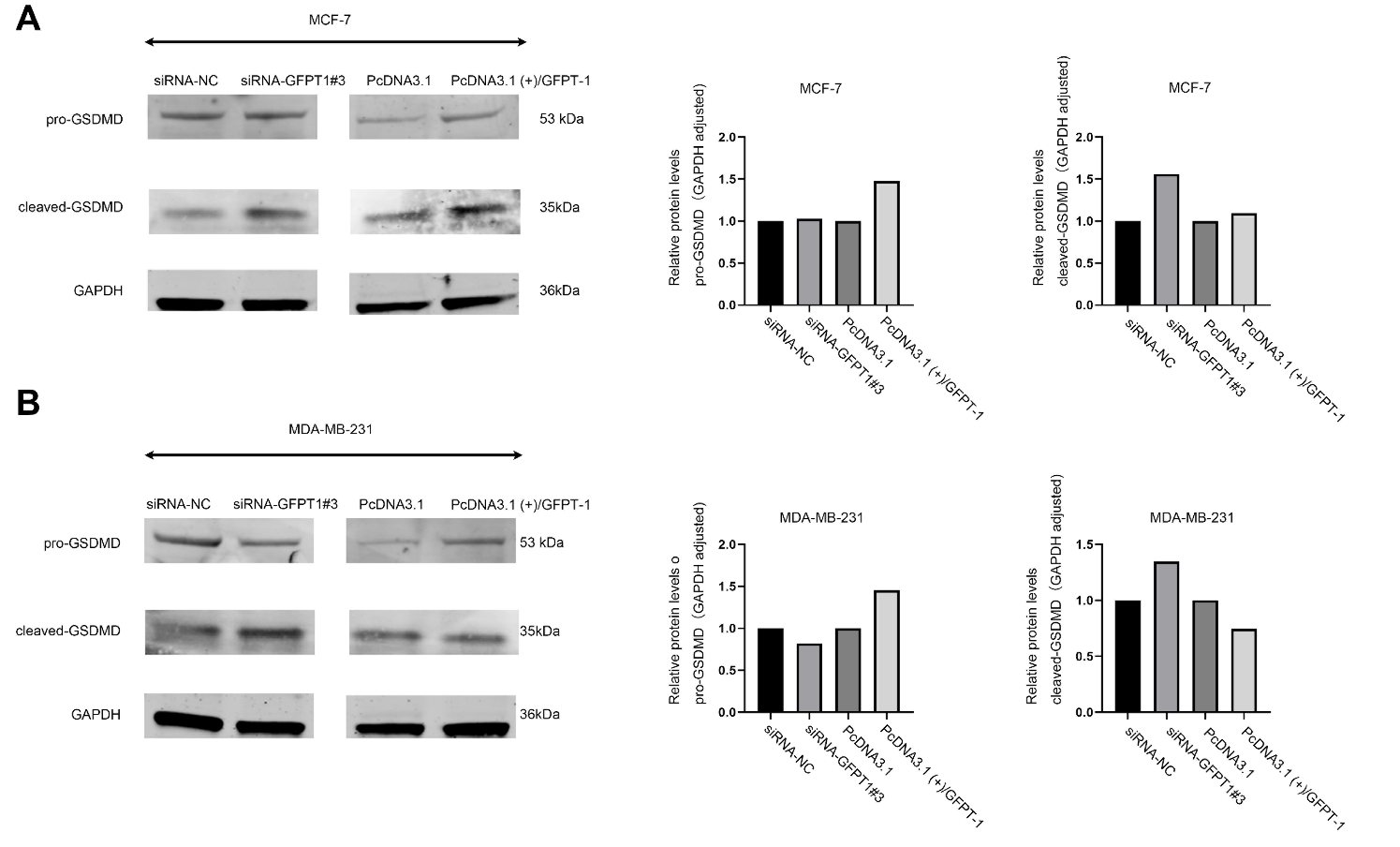


**Supplementary Figure 7.** Western blot analysis showed Pro-GSDMD and cleaved-GSDMD protein expression in MCF-7 (A) and MDA-MB-231 (B) cells after 48 hours of transfection with siRNA-NC, siRNA-GFPT1#3, PcDNA3.1, and PcDNA3.1(+)/GFPT1.

## Supplementary Table 1

| Symbol | logFC | AveExpr | t | P.Value | adj.P.Val | B | change |
| --- | --- | --- | --- | --- | --- | --- | --- |
| EIF2AK3 | 0.595011 | 5.161365 | 19.08392 | 1.05E-69 | 1.04E-65 | 147.5803 | UP |
| USO1 | 0.513244 | 6.803662 | 15.60439 | 2.08E-49 | 1.02E-45 | 101.3942 | UP |
| STRN | 0.524104 | 5.937767 | 15.54756 | 4.26E-49 | 1.68E-45 | 100.6843 | UP |
| COPA | 0.50064 | 8.858632 | 15.36936 | 4.01E-48 | 1.13E-44 | 98.4683 | UP |
| PGM3 | 0.512511 | 5.391958 | 14.99378 | 4.30E-46 | 1.06E-42 | 93.85037 | UP |
| SUCO | 0.645083 | 6.844283 | 14.95444 | 6.98E-46 | 1.53E-42 | 93.3709 | UP |
| SEC24A | 0.56911 | 5.534033 | 14.66738 | 2.35E-44 | 4.64E-41 | 89.89635 | UP |
| LMAN1 | 0.505671 | 7.600733 | 13.76237 | 1.15E-39 | 1.13E-36 | 79.23503 | UP |
| SELENOI | 0.532174 | 6.126037 | 13.75638 | 1.23E-39 | 1.13E-36 | 79.16597 | UP |
| PCYOX1 | 0.538994 | 7.070216 | 13.75157 | 1.30E-39 | 1.13E-36 | 79.11048 | UP |
| SEC24D | 0.624979 | 6.558152 | 13.37252 | 1.04E-37 | 6.85E-35 | 74.78531 | UP |
| GOLGA4 | 0.560586 | 7.241308 | 13.20949 | 6.68E-37 | 3.63E-34 | 72.95094 | UP |
| TBC1D8B | 0.596558 | 3.771582 | 12.90803 | 1.99E-35 | 8.01E-33 | 69.60119 | UP |
| MIGA1 | 0.587104 | 5.051219 | 12.82362 | 5.10E-35 | 2.01E-32 | 68.67326 | UP |
| VANGL1 | 0.516787 | 5.721055 | 12.56139 | 9.21E-34 | 3.24E-31 | 65.81841 | UP |
| ATP13A3 | 0.508778 | 6.821437 | 12.43061 | 3.84E-33 | 1.28E-30 | 64.41071 | UP |
| GPR180 | 0.521777 | 4.399493 | 12.36854 | 7.52E-33 | 2.43E-30 | 63.74638 | UP |
| KIF21A | 0.646033 | 4.753188 | 12.3529 | 8.91E-33 | 2.83E-30 | 63.57942 | UP |
| CCDC186 | 0.523769 | 5.257371 | 12.11584 | 1.14E-31 | 3.07E-29 | 61.06736 | UP |
| EDEM3 | 0.581653 | 6.548164 | 11.99903 | 3.94E-31 | 9.96E-29 | 59.84291 | UP |
| UHMK1 | 0.502196 | 7.744185 | 11.96061 | 5.91E-31 | 1.46E-28 | 59.44217 | UP |
| USP34 | 0.730728 | 6.298771 | 11.86996 | 1.54E-30 | 3.41E-28 | 58.50033 | UP |
| CNOT1 | 0.692928 | 6.991295 | 11.85292 | 1.84E-30 | 3.98E-28 | 58.32394 | UP |
| ZNF770 | 0.784235 | 4.759953 | 11.70067 | 9.02E-30 | 1.68E-27 | 56.75612 | UP |
| USP37 | 0.745693 | 3.740751 | 11.69772 | 9.30E-30 | 1.71E-27 | 56.72588 | UP |
| SECISBP2L | 0.514579 | 5.847307 | 11.65453 | 1.46E-29 | 2.52E-27 | 56.28409 | UP |
| RO60 | 0.506586 | 5.731039 | 11.63974 | 1.70E-29 | 2.86E-27 | 56.13301 | UP |
| DOCK7 | 0.503498 | 5.601673 | 11.50852 | 6.56E-29 | 9.81E-27 | 54.79951 | UP |
| NBEAL1 | 0.710858 | 3.643727 | 11.48259 | 8.56E-29 | 1.25E-26 | 54.53744 | UP |
| RAPH1 | 0.690687 | 2.945984 | 11.4522 | 1.17E-28 | 1.62E-26 | 54.23078 | UP |
| APOOL | 0.505582 | 4.745496 | 11.4211 | 1.61E-28 | 2.19E-26 | 53.91762 | UP |
| DYNC1LI2 | 0.586969 | 6.26621 | 11.39246 | 2.15E-28 | 2.85E-26 | 53.62978 | UP |
| ASAP2 | 0.692588 | 4.193954 | 11.35584 | 3.12E-28 | 4.05E-26 | 53.26263 | UP |
| ENPP4 | 0.736375 | 4.650775 | 11.29093 | 6.03E-28 | 7.58E-26 | 52.61408 | UP |
| HOOK1 | 0.565188 | 5.508627 | 11.14724 | 2.56E-27 | 2.89E-25 | 51.18867 | UP |
| HSPA13 | 0.506398 | 5.471844 | 11.14249 | 2.69E-27 | 3.01E-25 | 51.1418 | UP |
| ZBTB41 | 0.576088 | 5.998232 | 11.08535 | 4.76E-27 | 5.10E-25 | 50.57903 | UP |
| ASH1L | 0.538355 | 7.031053 | 10.97051 | 1.49E-26 | 1.51E-24 | 49.45509 | UP |
| ARFGEF2 | 0.546134 | 6.924385 | 10.95268 | 1.78E-26 | 1.77E-24 | 49.2814 | UP |
| UBE4A | 0.658459 | 5.071478 | 10.84616 | 5.07E-26 | 4.63E-24 | 48.24832 | UP |
| DENND4C | 0.563821 | 5.736267 | 10.78738 | 9.02E-26 | 7.67E-24 | 47.68174 | UP |
| MED13 | 0.528587 | 6.742675 | 10.77694 | 9.98E-26 | 8.42E-24 | 47.58135 | UP |
| GPD2 | 0.525071 | 6.422603 | 10.72202 | 1.70E-25 | 1.37E-23 | 47.05458 | UP |
| HLTF | 0.731293 | 4.963038 | 10.70959 | 1.92E-25 | 1.54E-23 | 46.93569 | UP |
| NOMO2 | 0.564828 | 3.815885 | 10.68546 | 2.43E-25 | 1.91E-23 | 46.70509 | UP |
| APAF1 | 0.511459 | 4.290258 | 10.66472 | 2.97E-25 | 2.30E-23 | 46.50731 | UP |
| HMGCS1 | 0.516185 | 5.672492 | 10.62838 | 4.22E-25 | 3.14E-23 | 46.16135 | UP |
| KLHL11 | 0.651871 | 2.851766 | 10.53556 | 1.03E-24 | 7.02E-23 | 45.28216 | UP |
| FMN1 | 0.705454 | 4.566653 | 10.22769 | 1.91E-23 | 1.03E-21 | 42.41049 | UP |
| HK2 | 0.565965 | 6.62385 | 10.21187 | 2.21E-23 | 1.19E-21 | 42.26482 | UP |
| ARHGAP5 | 0.722738 | 5.03229 | 10.16608 | 3.40E-23 | 1.74E-21 | 41.84413 | UP |
| AKAP9 | 0.567457 | 6.952779 | 10.06003 | 9.09E-23 | 4.34E-21 | 40.87574 | UP |
| RNF169 | 0.600975 | 4.306892 | 10.03058 | 1.19E-22 | 5.55E-21 | 40.60836 | UP |
| ECT2 | 0.541341 | 5.726174 | 9.973371 | 2.02E-22 | 8.89E-21 | 40.09065 | UP |
| LMBRD2 | 0.506196 | 5.010101 | 9.802387 | 9.60E-22 | 3.75E-20 | 38.55797 | UP |
| REL | 0.601614 | 3.942256 | 9.783078 | 1.14E-21 | 4.42E-20 | 38.38627 | UP |
| SEC24C | 0.55668 | 5.760058 | 9.718646 | 2.04E-21 | 7.48E-20 | 37.81533 | UP |
| SPIRE1 | 0.520094 | 4.652862 | 9.502933 | 1.40E-20 | 4.43E-19 | 35.92673 | UP |
| NFAT5 | 0.621715 | 5.120822 | 9.360661 | 4.86E-20 | 1.38E-18 | 34.70053 | UP |
| CEP295 | 0.537658 | 3.673367 | 9.353572 | 5.17E-20 | 1.45E-18 | 34.63985 | UP |
| CERS6 | 0.699154 | 6.047541 | 9.348734 | 5.40E-20 | 1.51E-18 | 34.59845 | UP |
| SMC4 | 0.618134 | 5.710354 | 9.346344 | 5.51E-20 | 1.54E-18 | 34.578 | UP |
| EPCAM | 0.502927 | 7.768627 | 9.301546 | 8.13E-20 | 2.21E-18 | 34.19561 | UP |
| SHPRH | 0.557458 | 3.597577 | 9.279506 | 9.84E-20 | 2.63E-18 | 34.00806 | UP |
| CMTM4 | 0.501774 | 5.994129 | 9.183209 | 2.26E-19 | 5.66E-18 | 33.19298 | UP |
| DNAJC6 | 0.677087 | 2.354644 | 9.154408 | 2.89E-19 | 7.11E-18 | 32.9506 | UP |
| CPD | 0.517359 | 7.333828 | 9.15215 | 2.95E-19 | 7.22E-18 | 32.93163 | UP |
| NOMO3 | 0.560065 | 2.646667 | 9.090742 | 4.98E-19 | 1.16E-17 | 32.4171 | UP |
| ARFGEF3 | 0.708245 | 6.897295 | 9.022797 | 8.86E-19 | 1.97E-17 | 31.85124 | UP |
| ANKRD18EP | 0.501661 | 2.867325 | 8.851181 | 3.74E-18 | 7.60E-17 | 30.43811 | UP |
| ATP1A1 | 0.528649 | 7.482048 | 8.704238 | 1.26E-17 | 2.32E-16 | 29.24665 | UP |
| GALNT7 | 0.638179 | 6.318437 | 8.675937 | 1.59E-17 | 2.86E-16 | 29.01914 | UP |
| DENND1B | 0.627417 | 5.447035 | 8.421314 | 1.24E-16 | 1.98E-15 | 27.00113 | UP |
| KDELR3 | 0.517762 | 5.154156 | 8.375595 | 1.79E-16 | 2.81E-15 | 26.64431 | UP |
| NUCB2 | 0.508757 | 7.106261 | 8.334493 | 2.48E-16 | 3.83E-15 | 26.32497 | UP |
| CDH1 | 0.800773 | 8.387271 | 8.320199 | 2.78E-16 | 4.26E-15 | 26.21424 | UP |
| KNL1 | 0.518831 | 3.735175 | 8.285919 | 3.64E-16 | 5.50E-15 | 25.94935 | UP |
| SHANK2 | 0.522955 | 5.532084 | 7.920317 | 6.14E-15 | 7.64E-14 | 23.18398 | UP |
| ENTPD5 | 0.550361 | 5.24774 | 7.400139 | 2.83E-13 | 2.67E-12 | 19.44043 | UP |
| GALNT3 | 0.758682 | 4.864454 | 7.392222 | 3.00E-13 | 2.82E-12 | 19.38521 | UP |
| RIMKLA | 0.571562 | 2.346645 | 7.313827 | 5.23E-13 | 4.72E-12 | 18.84128 | UP |
| COBL | 0.591544 | 5.273813 | 7.206652 | 1.11E-12 | 9.52E-12 | 18.10613 | UP |
| NEXMIF | 0.504412 | 2.066639 | 7.150186 | 1.65E-12 | 1.37E-11 | 17.72275 | UP |
| ANLN | 0.555636 | 5.080167 | 7.085605 | 2.57E-12 | 2.07E-11 | 17.28762 | UP |
| GRIA2 | 1.000441 | 1.671869 | 6.921839 | 7.85E-12 | 5.88E-11 | 16.20025 | UP |
| ASPM | 0.545449 | 4.666336 | 6.831257 | 1.44E-11 | 1.04E-10 | 15.60876 | UP |
| ENPP5 | 0.664067 | 5.151703 | 6.830835 | 1.45E-11 | 1.04E-10 | 15.60601 | UP |
| SLC7A11 | 0.525017 | 2.725388 | 6.799528 | 1.78E-11 | 1.27E-10 | 15.40324 | UP |
| FAM83B | 0.609771 | 2.427141 | 6.702187 | 3.38E-11 | 2.32E-10 | 14.77822 | UP |
| ANOS1 | 0.522739 | 4.467124 | 6.566098 | 8.20E-11 | 5.26E-10 | 13.91823 | UP |
| NRIP1 | 0.531789 | 6.379884 | 6.491161 | 1.32E-10 | 8.25E-10 | 13.45161 | UP |
| ME1 | 0.540562 | 3.821838 | 6.480395 | 1.42E-10 | 8.79E-10 | 13.38497 | UP |
| SLC2A10 | 0.504616 | 6.128872 | 6.468121 | 1.53E-10 | 9.46E-10 | 13.30913 | UP |
| PEX5L | 0.522157 | 1.155374 | 6.024109 | 2.37E-09 | 1.22E-08 | 10.65501 | UP |
| CEACAM5 | 0.964851 | 3.110421 | 5.962346 | 3.42E-09 | 1.72E-08 | 10.2997 | UP |
| PRKAA2 | 0.595979 | 3.472226 | 5.850118 | 6.60E-09 | 3.19E-08 | 9.66284 | UP |
| SLC9A2 | 0.575448 | 1.882446 | 5.618387 | 2.48E-08 | 1.10E-07 | 8.383689 | UP |
| TRIM36 | 0.506744 | 2.727956 | 5.554967 | 3.54E-08 | 1.54E-07 | 8.042073 | UP |
| TMEM178B | 0.512511 | 1.933783 | 5.272779 | 1.64E-07 | 6.48E-07 | 6.566416 | UP |
| SLC1A2 | 0.531017 | 3.400752 | 5.155172 | 3.04E-07 | 1.16E-06 | 5.972902 | UP |
| CPB1 | 1.271968 | 3.548744 | 5.093091 | 4.19E-07 | 1.56E-06 | 5.664727 | UP |
| PTPRN2 | 0.599517 | 3.321851 | 5.036514 | 5.60E-07 | 2.05E-06 | 5.386965 | UP |
| MUC5B | 0.865594 | 4.155733 | 4.957478 | 8.35E-07 | 2.98E-06 | 5.003888 | UP |
| CEACAM6 | 0.878257 | 4.810693 | 4.808238 | 1.75E-06 | 5.93E-06 | 4.296298 | UP |
| SLC30A8 | 0.747206 | 2.002358 | 4.556416 | 5.83E-06 | 1.81E-05 | 3.149272 | UP |
| KCNJ3 | 0.799325 | 2.652017 | 4.303106 | 1.85E-05 | 5.32E-05 | 2.055278 | UP |
| AARD | 0.520761 | 4.526847 | 4.154555 | 3.53E-05 | 9.72E-05 | 1.441783 | UP |
| BMPR1B | 0.811471 | 4.878466 | 4.142208 | 3.72E-05 | 0.000102 | 1.391729 | UP |
| KIF1A | 0.562181 | 1.879975 | 4.059129 | 5.30E-05 | 0.000142 | 1.058677 | UP |
| LRP2 | 0.642242 | 4.876147 | 3.748296 | 0.000188 | 0.000457 | -0.12935 | UP |
| SYT13 | 0.537987 | 3.730255 | 3.166627 | 0.001588 | 0.00326 | -2.10455 | UP |
| CCDC12 | -0.54297 | 4.911432 | -13.2796 | 3.01E-37 | 1.80E-34 | 73.73789 | DOWN |
| PDLIM7 | -0.59683 | 5.721156 | -12.3284 | 1.16E-32 | 3.63E-30 | 63.31864 | DOWN |
| RPS9 | -0.53044 | 9.131332 | -12.206 | 4.34E-32 | 1.26E-29 | 62.01811 | DOWN |
| INAFM1 | -0.54003 | 3.388845 | -12.0759 | 1.74E-31 | 4.52E-29 | 60.64736 | DOWN |
| NAT14 | -0.63301 | 4.287179 | -11.9328 | 7.93E-31 | 1.88E-28 | 59.15311 | DOWN |
| RPL28 | -0.54824 | 9.249529 | -11.8377 | 2.16E-30 | 4.57E-28 | 58.1665 | DOWN |
| BBC3 | -0.66053 | 3.579102 | -11.8242 | 2.48E-30 | 5.22E-28 | 58.02653 | DOWN |
| LYL1 | -0.56065 | 2.222427 | -11.7602 | 4.85E-30 | 9.86E-28 | 57.36721 | DOWN |
| CLEC11A | -0.77202 | 3.813805 | -11.6864 | 1.05E-29 | 1.86E-27 | 56.61038 | DOWN |
| NOP53 | -0.62376 | 7.730417 | -11.6658 | 1.29E-29 | 2.26E-27 | 56.39963 | DOWN |
| RPL35 | -0.52307 | 8.364741 | -11.5157 | 6.09E-29 | 9.18E-27 | 54.87249 | DOWN |
| RPS28 | -0.57672 | 7.591841 | -11.4641 | 1.04E-28 | 1.45E-26 | 54.35029 | DOWN |
| RPS15 | -0.54701 | 7.854454 | -11.4163 | 1.69E-28 | 2.28E-26 | 53.86972 | DOWN |
| CDC42EP5 | -0.62275 | 2.11714 | -11.1322 | 2.98E-27 | 3.30E-25 | 51.03996 | DOWN |
| CCDC85B | -0.75058 | 3.578539 | -11.0994 | 4.13E-27 | 4.48E-25 | 50.71725 | DOWN |
| NDUFA11 | -0.53027 | 2.823865 | -11.0664 | 5.75E-27 | 6.03E-25 | 50.39305 | DOWN |
| ANKS3 | -0.51507 | 4.091067 | -10.9992 | 1.12E-26 | 1.15E-24 | 49.73501 | DOWN |
| IFFO1 | -0.51204 | 3.30684 | -10.9664 | 1.55E-26 | 1.56E-24 | 49.41481 | DOWN |
| TSPAN4 | -0.52401 | 5.239692 | -10.9011 | 2.95E-26 | 2.83E-24 | 48.7804 | DOWN |
| TAMALIN | -0.55728 | 2.717966 | -10.8649 | 4.22E-26 | 3.95E-24 | 48.42946 | DOWN |
| SCRN2 | -0.58947 | 4.62511 | -10.8501 | 4.88E-26 | 4.50E-24 | 48.2866 | DOWN |
| ZNF428 | -0.56708 | 3.33749 | -10.7597 | 1.18E-25 | 9.79E-24 | 47.41585 | DOWN |
| ZNF688 | -0.51314 | 3.618637 | -10.7333 | 1.53E-25 | 1.26E-23 | 47.16226 | DOWN |
| ATP5F1D | -0.5957 | 5.349665 | -10.7284 | 1.60E-25 | 1.30E-23 | 47.11596 | DOWN |
| QTRT1 | -0.52097 | 4.860269 | -10.7049 | 2.01E-25 | 1.60E-23 | 46.89065 | DOWN |
| HEXD | -0.5173 | 4.305017 | -10.6368 | 3.89E-25 | 2.95E-23 | 46.24173 | DOWN |
| ANTKMT | -0.64096 | 3.594306 | -10.6286 | 4.21E-25 | 3.14E-23 | 46.1638 | DOWN |
| LSP1 | -0.63925 | 5.321611 | -10.5875 | 6.26E-25 | 4.51E-23 | 45.77356 | DOWN |
| RPLP2 | -0.50043 | 9.119204 | -10.5861 | 6.35E-25 | 4.54E-23 | 45.75966 | DOWN |
| RPL13AP25 | -0.53394 | 2.211519 | -10.5725 | 7.24E-25 | 5.12E-23 | 45.63145 | DOWN |
| CROCC | -0.58658 | 4.492642 | -10.5467 | 9.27E-25 | 6.37E-23 | 45.38764 | DOWN |
| PAXX | -0.54189 | 4.760456 | -10.5396 | 9.93E-25 | 6.78E-23 | 45.3203 | DOWN |
| CCDC124 | -0.53109 | 5.426322 | -10.528 | 1.11E-24 | 7.49E-23 | 45.21117 | DOWN |
| RARRES2 | -0.76333 | 5.062968 | -10.505 | 1.38E-24 | 9.09E-23 | 44.99405 | DOWN |
| IGFBP6 | -0.65861 | 3.113643 | -10.4427 | 2.50E-24 | 1.58E-22 | 44.40922 | DOWN |
| H1-10 | -0.52035 | 6.002127 | -10.3423 | 6.50E-24 | 3.86E-22 | 43.4712 | DOWN |
| ID3 | -0.53635 | 4.981647 | -10.3391 | 6.69E-24 | 3.96E-22 | 43.44205 | DOWN |
| RPL36 | -0.50918 | 8.453591 | -10.3175 | 8.21E-24 | 4.85E-22 | 43.24094 | DOWN |
| ZNF205 | -0.51778 | 3.560834 | -10.3148 | 8.42E-24 | 4.96E-22 | 43.21598 | DOWN |
| RPL13 | -0.50622 | 9.423251 | -10.2615 | 1.39E-23 | 7.90E-22 | 42.72263 | DOWN |
| RPS19 | -0.50301 | 9.350582 | -10.219 | 2.07E-23 | 1.12E-21 | 42.33042 | DOWN |
| NKD2 | -0.6843 | 2.844951 | -10.2137 | 2.18E-23 | 1.17E-21 | 42.28128 | DOWN |
| TPM2 | -0.71299 | 6.495059 | -10.2073 | 2.31E-23 | 1.22E-21 | 42.22316 | DOWN |
| REX1BD | -0.57694 | 4.041746 | -10.1299 | 4.76E-23 | 2.40E-21 | 41.51273 | DOWN |
| ARL4D | -0.57786 | 2.820628 | -10.0966 | 6.48E-23 | 3.19E-21 | 41.2089 | DOWN |
| TWIST2 | -0.5751 | 2.086881 | -10.0296 | 1.20E-22 | 5.59E-21 | 40.59923 | DOWN |
| JOSD2 | -0.54187 | 3.952483 | -10.0253 | 1.25E-22 | 5.77E-21 | 40.56032 | DOWN |
| HIC1 | -0.53845 | 3.288707 | -10.0199 | 1.32E-22 | 6.04E-21 | 40.51119 | DOWN |
| SCARF2 | -0.61474 | 3.3682 | -9.98538 | 1.81E-22 | 8.03E-21 | 40.19913 | DOWN |
| TNFRSF4 | -0.52389 | 2.062615 | -9.95767 | 2.33E-22 | 1.01E-20 | 39.949 | DOWN |
| GADD45GIP1 | -0.5314 | 5.161109 | -9.93128 | 2.97E-22 | 1.26E-20 | 39.71135 | DOWN |
| PCOLCE | -0.59258 | 5.95446 | -9.88993 | 4.33E-22 | 1.78E-20 | 39.34 | DOWN |
| PLEKHA4 | -0.70957 | 4.706488 | -9.86635 | 5.37E-22 | 2.18E-20 | 39.1288 | DOWN |
| CLDN5 | -0.75475 | 3.005989 | -9.83951 | 6.85E-22 | 2.73E-20 | 38.88887 | DOWN |
| PRRX2 | -0.60182 | 2.901758 | -9.82453 | 7.85E-22 | 3.11E-20 | 38.75526 | DOWN |
| PLAC9 | -0.63586 | 2.156185 | -9.79541 | 1.02E-21 | 3.98E-20 | 38.49594 | DOWN |
| WDR86 | -0.56189 | 1.651475 | -9.78265 | 1.15E-21 | 4.43E-20 | 38.38246 | DOWN |
| GADD45B | -0.54022 | 5.758571 | -9.76269 | 1.37E-21 | 5.26E-20 | 38.20524 | DOWN |
| LGALS1 | -0.5207 | 8.140148 | -9.75873 | 1.42E-21 | 5.44E-20 | 38.17018 | DOWN |
| EMILIN1 | -0.72295 | 6.047698 | -9.73028 | 1.84E-21 | 6.82E-20 | 37.91817 | DOWN |
| SEPTIN1 | -0.52022 | 2.776154 | -9.72952 | 1.85E-21 | 6.86E-20 | 37.9115 | DOWN |
| GSDMD | -0.55834 | 6.147658 | -9.72064 | 2.01E-21 | 7.37E-20 | 37.83295 | DOWN |
| TGFB1I1 | -0.51068 | 4.739894 | -9.71917 | 2.03E-21 | 7.46E-20 | 37.81998 | DOWN |
| CCDC88B | -0.63306 | 3.455086 | -9.67951 | 2.90E-21 | 1.03E-19 | 37.4701 | DOWN |
| CAVIN3 | -0.52635 | 2.027645 | -9.67014 | 3.16E-21 | 1.10E-19 | 37.38758 | DOWN |
| FSTL3 | -0.59553 | 4.399952 | -9.55929 | 8.48E-21 | 2.78E-19 | 36.41671 | DOWN |
| AC005912.1 | -0.57651 | 2.407497 | -9.51292 | 1.28E-20 | 4.07E-19 | 36.01342 | DOWN |
| TEDC1 | -0.52619 | 3.293912 | -9.48347 | 1.66E-20 | 5.16E-19 | 35.75805 | DOWN |
| RPL3P4 | -0.63763 | 3.261206 | -9.46111 | 2.02E-20 | 6.17E-19 | 35.56466 | DOWN |
| MXRA8 | -0.66982 | 6.752667 | -9.417 | 2.97E-20 | 8.83E-19 | 35.18427 | DOWN |
| ARHGAP4 | -0.57353 | 4.341604 | -9.37982 | 4.11E-20 | 1.18E-18 | 34.86472 | DOWN |
| ADAM33 | -0.67553 | 2.10029 | -9.352 | 5.24E-20 | 1.47E-18 | 34.62642 | DOWN |
| CEBPD | -0.69499 | 4.930765 | -9.35059 | 5.31E-20 | 1.49E-18 | 34.61431 | DOWN |
| ARHGAP45 | -0.52583 | 4.9922 | -9.31513 | 7.23E-20 | 1.99E-18 | 34.31136 | DOWN |
| ZNF219 | -0.54855 | 4.146674 | -9.24206 | 1.36E-19 | 3.55E-18 | 33.69023 | DOWN |
| CPXM1 | -0.75009 | 4.6682 | -9.21331 | 1.74E-19 | 4.46E-18 | 33.44703 | DOWN |
| PODNL1 | -0.60835 | 3.318466 | -9.16786 | 2.58E-19 | 6.36E-18 | 33.06372 | DOWN |
| MIB2 | -0.50273 | 4.232931 | -9.14514 | 3.13E-19 | 7.61E-18 | 32.87275 | DOWN |
| RPL21P16 | -0.52514 | 1.485937 | -9.13582 | 3.39E-19 | 8.23E-18 | 32.79449 | DOWN |
| PSTPIP1 | -0.58055 | 2.313203 | -9.11768 | 3.96E-19 | 9.45E-18 | 32.64242 | DOWN |
| GZMM | -0.50678 | 1.144952 | -9.09149 | 4.94E-19 | 1.15E-17 | 32.42334 | DOWN |
| IER2 | -0.53113 | 5.917621 | -9.0721 | 5.83E-19 | 1.34E-17 | 32.26149 | DOWN |
| MSC | -0.5056 | 2.700596 | -9.03601 | 7.92E-19 | 1.78E-17 | 31.96097 | DOWN |
| RRAD | -0.54054 | 1.253699 | -9.02197 | 8.92E-19 | 1.99E-17 | 31.84437 | DOWN |
| ID1 | -0.55869 | 3.485145 | -9.01473 | 9.48E-19 | 2.10E-17 | 31.78428 | DOWN |
| LTB | -0.83119 | 2.591135 | -8.93756 | 1.82E-18 | 3.91E-17 | 31.1465 | DOWN |
| RASAL3 | -0.55253 | 3.234541 | -8.90621 | 2.36E-18 | 4.99E-17 | 30.88872 | DOWN |
| COL7A1 | -0.73792 | 3.942998 | -8.90602 | 2.37E-18 | 5.00E-17 | 30.88716 | DOWN |
| NDN | -0.51902 | 3.646006 | -8.89286 | 2.64E-18 | 5.56E-17 | 30.77914 | DOWN |
| RPL7P9 | -0.51913 | 2.751625 | -8.88698 | 2.77E-18 | 5.81E-17 | 30.73099 | DOWN |
| LOXL1 | -0.54493 | 5.587359 | -8.86462 | 3.34E-18 | 6.85E-17 | 30.54792 | DOWN |
| APOE | -0.70669 | 7.573186 | -8.85666 | 3.57E-18 | 7.29E-17 | 30.4829 | DOWN |
| CCDC8 | -0.6777 | 3.59819 | -8.83021 | 4.45E-18 | 8.97E-17 | 30.26699 | DOWN |
| CORO1A | -0.6364 | 5.143179 | -8.82013 | 4.84E-18 | 9.66E-17 | 30.18492 | DOWN |
| CFD | -0.9227 | 3.954452 | -8.81445 | 5.07E-18 | 1.01E-16 | 30.13869 | DOWN |
| BATF | -0.59365 | 3.56121 | -8.79186 | 6.12E-18 | 1.20E-16 | 29.95502 | DOWN |
| DUX4L50 | -0.66816 | 1.733455 | -8.78274 | 6.60E-18 | 1.29E-16 | 29.88101 | DOWN |
| COL16A1 | -0.62055 | 6.413913 | -8.77195 | 7.21E-18 | 1.40E-16 | 29.79354 | DOWN |
| IFITM3 | -0.53103 | 8.562908 | -8.49795 | 6.73E-17 | 1.12E-15 | 27.60304 | DOWN |
| RCN3 | -0.57307 | 5.292548 | -8.46631 | 8.68E-17 | 1.42E-15 | 27.35397 | DOWN |
| GGT5 | -0.52184 | 5.23967 | -8.45706 | 9.35E-17 | 1.52E-15 | 27.28126 | DOWN |
| ACAP1 | -0.57613 | 3.222521 | -8.44892 | 9.98E-17 | 1.61E-15 | 27.21742 | DOWN |
| MRPL12 | -0.52688 | 2.162128 | -8.41648 | 1.29E-16 | 2.06E-15 | 26.96336 | DOWN |
| NNMT | -0.55495 | 6.233671 | -8.35431 | 2.12E-16 | 3.30E-15 | 26.47875 | DOWN |
| GAS6 | -0.54681 | 6.035677 | -8.34557 | 2.27E-16 | 3.52E-15 | 26.41092 | DOWN |
| PDLIM4 | -0.58416 | 3.961645 | -8.2893 | 3.55E-16 | 5.36E-15 | 25.9754 | DOWN |
| NME3 | -0.55528 | 5.476175 | -8.28519 | 3.66E-16 | 5.53E-15 | 25.9437 | DOWN |
| C1QTNF1 | -0.50417 | 4.666809 | -8.2251 | 5.87E-16 | 8.55E-15 | 25.48175 | DOWN |
| COL6A2 | -0.58131 | 9.448222 | -8.22229 | 6.00E-16 | 8.73E-15 | 25.46021 | DOWN |
| LIMD2 | -0.58085 | 4.00363 | -8.19384 | 7.50E-16 | 1.08E-14 | 25.24259 | DOWN |
| PHLDA3 | -0.51322 | 5.310582 | -8.15452 | 1.02E-15 | 1.43E-14 | 24.94286 | DOWN |
| NOXA1 | -0.56805 | 3.50114 | -8.14874 | 1.06E-15 | 1.49E-14 | 24.89893 | DOWN |
| TWIST1 | -0.53662 | 2.849563 | -8.133 | 1.20E-15 | 1.67E-14 | 24.77933 | DOWN |
| RASGRP2 | -0.52071 | 2.176889 | -8.1229 | 1.30E-15 | 1.80E-14 | 24.70278 | DOWN |
| SCNN1D | -0.50342 | 2.152118 | -8.1172 | 1.36E-15 | 1.87E-14 | 24.65955 | DOWN |
| KIFC2 | -0.58506 | 4.094481 | -8.10906 | 1.45E-15 | 1.99E-14 | 24.59793 | DOWN |
| EBF4 | -0.62798 | 4.155072 | -8.03905 | 2.48E-15 | 3.31E-14 | 24.07004 | DOWN |
| PALM | -0.61601 | 4.762019 | -8.03478 | 2.57E-15 | 3.40E-14 | 24.03798 | DOWN |
| TBC1D10C | -0.557 | 2.839314 | -7.98049 | 3.89E-15 | 4.99E-14 | 23.63159 | DOWN |
| MFAP4 | -0.81573 | 5.01166 | -7.95059 | 4.88E-15 | 6.16E-14 | 23.40882 | DOWN |
| RHPN1 | -0.54513 | 5.319623 | -7.94615 | 5.05E-15 | 6.35E-14 | 23.37576 | DOWN |
| COL6A1 | -0.56124 | 9.311972 | -7.90509 | 6.89E-15 | 8.51E-14 | 23.07116 | DOWN |
| HAGHL | -0.57662 | 3.32488 | -7.87776 | 8.47E-15 | 1.03E-13 | 22.8692 | DOWN |
| C1R | -0.51845 | 7.528909 | -7.85905 | 9.76E-15 | 1.17E-13 | 22.73134 | DOWN |
| MEGF6 | -0.53578 | 4.875909 | -7.84397 | 1.09E-14 | 1.30E-13 | 22.62043 | DOWN |
| TNFRSF12A | -0.52721 | 5.344023 | -7.78528 | 1.70E-14 | 1.96E-13 | 22.19041 | DOWN |
| SPHK1 | -0.52178 | 4.591814 | -7.75592 | 2.11E-14 | 2.39E-13 | 21.97646 | DOWN |
| CTXN1 | -0.55112 | 4.388638 | -7.63996 | 4.98E-14 | 5.28E-13 | 21.13821 | DOWN |
| CYBA | -0.54795 | 5.718807 | -7.63968 | 4.99E-14 | 5.28E-13 | 21.13621 | DOWN |
| TCF7 | -0.5244 | 3.969918 | -7.63739 | 5.07E-14 | 5.37E-13 | 21.11976 | DOWN |
| ISLR | -0.53731 | 6.981749 | -7.6218 | 5.69E-14 | 5.98E-13 | 21.00797 | DOWN |
| ACKR1 | -0.91925 | 3.519424 | -7.60405 | 6.48E-14 | 6.73E-13 | 20.88089 | DOWN |
| ZAP70 | -0.59376 | 2.242772 | -7.60127 | 6.61E-14 | 6.85E-13 | 20.86106 | DOWN |
| HES4 | -0.50226 | 2.69865 | -7.57948 | 7.75E-14 | 7.94E-13 | 20.70547 | DOWN |
| HLA-DPB1 | -0.507 | 7.281636 | -7.57662 | 7.91E-14 | 8.09E-13 | 20.68509 | DOWN |
| MATK | -0.52064 | 2.006674 | -7.53497 | 1.07E-13 | 1.08E-12 | 20.38895 | DOWN |
| TPSAB1 | -0.72386 | 3.096899 | -7.50905 | 1.29E-13 | 1.29E-12 | 20.20541 | DOWN |
| PLD4 | -0.55369 | 2.718328 | -7.48908 | 1.49E-13 | 1.47E-12 | 20.06445 | DOWN |
| NBL1 | -0.55112 | 6.964803 | -7.46821 | 1.74E-13 | 1.70E-12 | 19.91742 | DOWN |
| MAOB | -0.99921 | 5.335279 | -7.46662 | 1.76E-13 | 1.71E-12 | 19.9062 | DOWN |
| APOBR | -0.51818 | 3.769875 | -7.45922 | 1.85E-13 | 1.80E-12 | 19.85421 | DOWN |
| DPT | -0.61827 | 3.029997 | -7.45416 | 1.92E-13 | 1.86E-12 | 19.81863 | DOWN |
| TRBC1 | -0.64998 | 2.835609 | -7.42509 | 2.37E-13 | 2.27E-12 | 19.61483 | DOWN |
| GALNT16 | -0.69743 | 4.046097 | -7.42034 | 2.45E-13 | 2.34E-12 | 19.58157 | DOWN |
| GADD45G | -0.56616 | 3.391458 | -7.40003 | 2.83E-13 | 2.68E-12 | 19.43968 | DOWN |
| CNN1 | -0.62856 | 3.875769 | -7.33401 | 4.53E-13 | 4.15E-12 | 18.98079 | DOWN |
| CD52 | -0.61145 | 3.502362 | -7.25981 | 7.66E-13 | 6.75E-12 | 18.46954 | DOWN |
| CCDC78 | -0.5787 | 2.599688 | -7.25487 | 7.93E-13 | 6.98E-12 | 18.43565 | DOWN |
| CCL19 | -0.88131 | 3.153879 | -7.22704 | 9.63E-13 | 8.34E-12 | 18.2452 | DOWN |
| BMERB1 | -0.63496 | 4.277974 | -7.17345 | 1.40E-12 | 1.18E-11 | 17.88036 | DOWN |
| PTN | -0.73947 | 3.647837 | -7.14132 | 1.75E-12 | 1.45E-11 | 17.66277 | DOWN |
| MMP2 | -0.56147 | 8.400123 | -7.12292 | 1.99E-12 | 1.63E-11 | 17.53857 | DOWN |
| HTRA1 | -0.50303 | 7.68468 | -7.11116 | 2.16E-12 | 1.76E-11 | 17.45936 | DOWN |
| TRBC2 | -0.65209 | 3.451281 | -7.07364 | 2.79E-12 | 2.24E-11 | 17.20739 | DOWN |
| KLK4 | -0.50737 | 1.695639 | -7.06327 | 3.00E-12 | 2.39E-11 | 17.13793 | DOWN |
| TFAP2B | -1.29227 | 4.157064 | -7.04663 | 3.36E-12 | 2.66E-11 | 17.02674 | DOWN |
| CITED4 | -0.7471 | 4.893986 | -7.0209 | 4.01E-12 | 3.13E-11 | 16.8552 | DOWN |
| CORO6 | -0.7072 | 2.025159 | -7.01268 | 4.24E-12 | 3.31E-11 | 16.80058 | DOWN |
| CD7 | -0.52139 | 2.215337 | -6.99411 | 4.81E-12 | 3.73E-11 | 16.67729 | DOWN |
| NAPSB | -0.5054 | 3.262073 | -6.94729 | 6.61E-12 | 5.01E-11 | 16.3677 | DOWN |
| JAML | -0.50697 | 2.955167 | -6.86166 | 1.18E-11 | 8.58E-11 | 15.80652 | DOWN |
| MEG3 | -0.55437 | 3.530167 | -6.8275 | 1.48E-11 | 1.06E-10 | 15.58439 | DOWN |
| TPSB2 | -0.72787 | 3.392733 | -6.794 | 1.85E-11 | 1.31E-10 | 15.36756 | DOWN |
| ADRA2A | -0.59057 | 4.027047 | -6.69118 | 3.64E-11 | 2.47E-10 | 14.70804 | DOWN |
| PYCARD | -0.51541 | 4.585869 | -6.68917 | 3.69E-11 | 2.50E-10 | 14.69528 | DOWN |
| CD3D | -0.53044 | 2.364244 | -6.6768 | 4.00E-11 | 2.70E-10 | 14.61657 | DOWN |
| NGFR | -0.65018 | 3.191026 | -6.64884 | 4.80E-11 | 3.21E-10 | 14.43917 | DOWN |
| HTRA3 | -0.58675 | 6.244316 | -6.6188 | 5.83E-11 | 3.84E-10 | 14.24933 | DOWN |
| HLA-DQB1 | -0.53294 | 6.253506 | -6.57079 | 7.95E-11 | 5.12E-10 | 13.94763 | DOWN |
| CYP4F23P | -0.59248 | 2.35502 | -6.51781 | 1.12E-10 | 7.03E-10 | 13.61699 | DOWN |
| CCN5 | -0.64778 | 4.067826 | -6.47731 | 1.45E-10 | 8.95E-10 | 13.36591 | DOWN |
| CILP2 | -0.51934 | 3.686336 | -6.43099 | 1.94E-10 | 1.18E-09 | 13.08049 | DOWN |
| GRP | -0.51688 | 2.026949 | -6.36081 | 3.02E-10 | 1.78E-09 | 12.65166 | DOWN |
| ETNK2 | -0.52845 | 4.546532 | -6.34185 | 3.40E-10 | 1.99E-09 | 12.53657 | DOWN |
| FST | -0.54825 | 3.680469 | -6.2565 | 5.78E-10 | 3.27E-09 | 12.02237 | DOWN |
| CCL21 | -0.70137 | 2.643969 | -6.25173 | 5.95E-10 | 3.37E-09 | 11.99383 | DOWN |
| MIR205HG | -0.72389 | 3.486614 | -6.22693 | 6.93E-10 | 3.87E-09 | 11.84572 | DOWN |
| COL1A1 | -0.55057 | 12.2466 | -6.20603 | 7.88E-10 | 4.37E-09 | 11.72129 | DOWN |
| SNCG | -0.64187 | 3.51839 | -6.06775 | 1.82E-09 | 9.50E-09 | 10.90814 | DOWN |
| SHISA2 | -0.83959 | 3.988919 | -6.01405 | 2.51E-09 | 1.29E-08 | 10.59689 | DOWN |
| WNT4 | -0.52252 | 3.044561 | -6.00155 | 2.71E-09 | 1.38E-08 | 10.52483 | DOWN |
| KRT14 | -1.05103 | 4.851959 | -5.97723 | 3.13E-09 | 1.58E-08 | 10.38501 | DOWN |
| CXCL14 | -0.87055 | 6.525245 | -5.97345 | 3.20E-09 | 1.61E-08 | 10.3633 | DOWN |
| METRN | -0.53712 | 5.247085 | -5.96325 | 3.40E-09 | 1.71E-08 | 10.30487 | DOWN |
| PHYHD1 | -0.63319 | 3.493008 | -5.95888 | 3.49E-09 | 1.75E-08 | 10.27987 | DOWN |
| DEGS2 | -0.74295 | 5.401545 | -5.94409 | 3.81E-09 | 1.90E-08 | 10.19536 | DOWN |
| PLAAT4 | -0.57113 | 6.368131 | -5.90491 | 4.80E-09 | 2.36E-08 | 9.972334 | DOWN |
| PNMT | -0.5817 | 1.280448 | -5.83529 | 7.20E-09 | 3.45E-08 | 9.57952 | DOWN |
| ELN | -0.63471 | 6.018972 | -5.76492 | 1.08E-08 | 5.05E-08 | 9.186929 | DOWN |
| SFRP2 | -0.57448 | 8.431765 | -5.74005 | 1.25E-08 | 5.78E-08 | 9.049206 | DOWN |
| CD3E | -0.53526 | 3.577923 | -5.70673 | 1.51E-08 | 6.92E-08 | 8.865646 | DOWN |
| HOXC13 | -0.52166 | 3.024845 | -5.671 | 1.85E-08 | 8.37E-08 | 8.669878 | DOWN |
| CPNE7 | -0.51055 | 2.77973 | -5.62948 | 2.33E-08 | 1.04E-07 | 8.443793 | DOWN |
| G0S2 | -0.5413 | 2.917452 | -5.62024 | 2.46E-08 | 1.09E-07 | 8.39373 | DOWN |
| IRX1 | -0.56751 | 2.015554 | -5.60382 | 2.69E-08 | 1.19E-07 | 8.304877 | DOWN |
| PGLYRP2 | -0.61172 | 1.856584 | -5.59489 | 2.83E-08 | 1.25E-07 | 8.25667 | DOWN |
| PWWP3B | -0.53539 | 2.348924 | -5.5925 | 2.87E-08 | 1.26E-07 | 8.243797 | DOWN |
| CYP4F8 | -0.73195 | 1.564384 | -5.58305 | 3.03E-08 | 1.33E-07 | 8.1929 | DOWN |
| RBP1 | -0.59989 | 4.601014 | -5.57733 | 3.12E-08 | 1.37E-07 | 8.162104 | DOWN |
| COL17A1 | -0.76655 | 3.829846 | -5.57529 | 3.16E-08 | 1.38E-07 | 8.151155 | DOWN |
| BST2 | -0.51094 | 7.258214 | -5.53055 | 4.05E-08 | 1.75E-07 | 7.911528 | DOWN |
| COMP | -0.64423 | 6.239249 | -5.51488 | 4.42E-08 | 1.89E-07 | 7.828034 | DOWN |
| RAI2 | -0.52129 | 4.153144 | -5.47485 | 5.51E-08 | 2.33E-07 | 7.615755 | DOWN |
| CYP4F22 | -0.63186 | 2.150586 | -5.38707 | 8.88E-08 | 3.66E-07 | 7.155341 | DOWN |
| FABP4 | -0.81635 | 3.972221 | -5.37425 | 9.52E-08 | 3.90E-07 | 7.088673 | DOWN |
| PGGHG | -0.55446 | 5.346723 | -5.34824 | 1.10E-07 | 4.45E-07 | 6.953907 | DOWN |
| KRT17 | -0.88955 | 5.37138 | -5.3155 | 1.31E-07 | 5.24E-07 | 6.785151 | DOWN |
| BMP7 | -0.53093 | 2.650274 | -5.26957 | 1.67E-07 | 6.59E-07 | 6.550062 | DOWN |
| SUSD3 | -0.65815 | 4.913477 | -5.20969 | 2.29E-07 | 8.87E-07 | 6.246463 | DOWN |
| SEC14L2 | -0.54053 | 5.038463 | -5.04681 | 5.31E-07 | 1.95E-06 | 5.437308 | DOWN |
| ZBTB16 | -0.55764 | 2.770557 | -4.98498 | 7.27E-07 | 2.62E-06 | 5.136549 | DOWN |
| CHIT1 | -0.57432 | 2.360608 | -4.8531 | 1.40E-06 | 4.83E-06 | 4.506812 | DOWN |
| SAA1 | -0.63543 | 3.978366 | -4.84446 | 1.47E-06 | 5.03E-06 | 4.466134 | DOWN |
| ARHGAP40 | -0.64333 | 2.216139 | -4.84048 | 1.49E-06 | 5.12E-06 | 4.447436 | DOWN |
| CILP | -0.5908 | 6.419117 | -4.79622 | 1.86E-06 | 6.27E-06 | 4.240236 | DOWN |
| IGHA2 | -0.75101 | 5.484665 | -4.78578 | 1.95E-06 | 6.57E-06 | 4.191621 | DOWN |
| TCIM | -0.52012 | 5.27177 | -4.73072 | 2.55E-06 | 8.43E-06 | 3.936917 | DOWN |
| TP63 | -0.51965 | 3.045692 | -4.69902 | 2.97E-06 | 9.71E-06 | 3.791556 | DOWN |
| RPS28P7 | -0.58003 | 5.143225 | -4.66559 | 3.48E-06 | 1.12E-05 | 3.639316 | DOWN |
| DHRS2 | -0.89215 | 3.775352 | -4.64178 | 3.90E-06 | 1.25E-05 | 3.531458 | DOWN |
| SCGB3A1 | -0.52991 | 1.53261 | -4.6394 | 3.95E-06 | 1.26E-05 | 3.520735 | DOWN |
| KRT5 | -0.81069 | 5.430878 | -4.45039 | 9.51E-06 | 2.87E-05 | 2.684028 | DOWN |
| PLIN4 | -0.62714 | 4.224879 | -4.4502 | 9.52E-06 | 2.88E-05 | 2.683208 | DOWN |
| KRT7 | -0.58262 | 7.393759 | -4.4479 | 9.62E-06 | 2.90E-05 | 2.673236 | DOWN |
| WNK4 | -0.56521 | 3.480019 | -4.27099 | 2.13E-05 | 6.07E-05 | 1.920872 | DOWN |
| AREG | -0.59476 | 3.870176 | -4.24256 | 2.41E-05 | 6.80E-05 | 1.802701 | DOWN |
| APOD | -0.61997 | 6.796773 | -4.21352 | 2.74E-05 | 7.65E-05 | 1.682829 | DOWN |
| KRT15 | -0.65305 | 5.550391 | -4.19627 | 2.95E-05 | 8.21E-05 | 1.611964 | DOWN |
| SPTSSB | -0.52612 | 3.389661 | -4.06958 | 5.07E-05 | 0.000136 | 1.10022 | DOWN |
| IGHA1 | -0.6256 | 7.921705 | -3.9549 | 8.18E-05 | 0.000212 | 0.650082 | DOWN |
| PLIN1 | -0.53368 | 3.363135 | -3.93471 | 8.89E-05 | 0.000229 | 0.572133 | DOWN |
| IGHG4 | -0.5599 | 5.635968 | -3.84367 | 0.000129 | 0.000322 | 0.225399 | DOWN |
| ADH1B | -0.59198 | 3.457581 | -3.75908 | 0.00018 | 0.000439 | -0.08966 | DOWN |
| MTND1P23 | -0.50766 | 1.331283 | -3.63948 | 0.000287 | 0.000676 | -0.5235 | DOWN |
| STC2 | -0.52993 | 7.513661 | -3.61126 | 0.000319 | 0.000746 | -0.62389 | DOWN |
| CLIC6 | -0.60751 | 4.004065 | -3.54825 | 0.000405 | 0.000929 | -0.84524 | DOWN |
| CXCL13 | -0.51814 | 3.184487 | -3.53716 | 0.000423 | 0.000966 | -0.88383 | DOWN |
| SERPINA5 | -0.50937 | 4.450803 | -3.42026 | 0.00065 | 0.001433 | -1.28321 | DOWN |
| LINC00993 | -0.56819 | 4.103094 | -3.19306 | 0.001451 | 0.002998 | -2.02184 | DOWN |
| PTPRT | -0.51053 | 4.388635 | -3.1813 | 0.00151 | 0.003112 | -2.05871 | DOWN |
| PPP1R1B | -0.51974 | 4.790307 | -3.12233 | 0.001845 | 0.003747 | -2.24163 | DOWN |
| PGR | -0.56388 | 5.059682 | -3.0217 | 0.002576 | 0.005089 | -2.54604 | DOWN |
| CYP4Z1 | -0.50593 | 3.309492 | -2.72619 | 0.006516 | 0.01187 | -3.38321 | DOWN |
